# Effects of growth promoting microorganisms on tomato seedlings growing in different media conditions

**DOI:** 10.1371/journal.pone.0259380

**Published:** 2021-11-03

**Authors:** Robert Pokluda, Lucia Ragasová, Miloš Jurica, Andrzej Kalisz, Monika Komorowska, Marcin Niemiec, Agnieszka Sekara

**Affiliations:** 1 Faculty of Horticulture, Department of Vegetable Sciences and Floriculture, Mendel University in Brno, Brno, Czech Republic; 2 Faculty of Biotechnology and Horticulture, Department of Horticulture, University of Agriculture in Krakow, Krakow, Poland; 3 Faculty of Agriculture and Economics, Department of Agricultural and Environmental Chemistry, University of Agriculture in Krakow, Krakow, Poland; Bangabandhu Sheikh Mujibur Rahman Agricultural University, BANGLADESH

## Abstract

Plant growth-promoting microbes (PGPM) play vital roles in maintaining crop fitness and soil health in stressed environments. Research have included analysis-based cultivation of soil-microbial-plant relationships to clarify microbiota potential. The goal of the research was to (i) evaluate the symbiotic microorganism effects on tomato seedling fitness under stressed conditions simulating a fragile soil susceptible to degradation; (ii) compare the plant-microbial interactions after inoculation with microbial isolates and fungi-bacteria consortia; (iii) develop an effective crop-microbial network, which improves soil and plant status. The experimental design included non-inoculated treatments with peat and sand at ratios of 50:50, 70:30, 100:0 (v:v), inoculated treatments with arbuscular mycorrhizal fungi (AMF) and *Azospirillum brasilense* (AZ) using the aforementioned peat:sand ratios; and treatment with peat co-inoculated with AMF and *Saccharothrix tamanrassetensis* (S). AMF + AZ increased root fresh weight in peat substrate compared to the control (4.4 to 3.3 g plant^–1^). An increase in shoot fresh weight was detected in the AMF + AZ treatment with a 50:50 peat:sand ratio (10.1 to 8.5 g plant^-1^). AMF + AZ reduced antioxidant activity (DPPH) (18–34%) in leaves, whereas AMF + S had the highest DPPH in leaves and roots (45%). Total leaf phenolic content was higher in control with a decreased proportion of peat. Peroxidase activity was enhanced in AMF + AZ and AMF + S treatments, except for AMF + AZ in peat. Microscopic root assays revealed the ability of AMF to establish strong fungal-tomato symbiosis; the colonization rate was 78–89%. AMF + AZ accelerated K and Mg accumulation in tomato leaves in treatments reflecting soil stress. To date, there has been no relevant information regarding the successful AMF and *Saccharothrix* co-inoculation relationship. This study confirmed that AMF + S could increase the P, S, and Fe status of seedlings under high organic C content conditions. The improved tomato growth and nutrient acquisition demonstrated the potential of PGPM colonization under degraded soil conditions.

## Introduction

The predicted and persistent threat of soil degradation driven by climatic and anthropogenic forces necessitates the development of strict directives for the protection of soils, as recently promoted by the European Union [1]. Erosion, and the consequent decline in organic soil matter, reduces overall soil biomass and biological activity. Ultimately, it profoundly affects the diversity of microbes coexisting in the soil ecosystem [2,3]. Understanding the soil microbial communities and their beneficial roles in agricultural land is crucial to maintaining plant fitness and preventing soil degradation [4–8]. The rhizosphere is home to many interactions that substantially affect soil, microorganisms, and plants. In agricultural practices, root-associated arbuscular mycorrhizal fungi AMF application is highly effective, ultimately resulting in the improvement of crop growth, health, yield, and general fitness [9–11]. Nutrient availability, especially phosphorus (P), is essential for multiple processes. P is delivered as phosphate to the root through AMF specialized structures called arbuscules, formed within cortical cells [12]. Volatile compounds released by rhizosphere microorganisms are also reported to be signal molecules stimulating lateral root development [13]. Fungi release carbolic acid, and low-molecular-weight organic acids, that bind to metal ions in the soil solution, including Al, Ca, Cu, Fe, Mg, Mn, and Zn ions, promoting mineral weathering and enabling their absorption by hyphae and transport to plant roots [12]. Enhanced nutrient uptake, mainly that of N and P, is rewarded with C compounds derived from host plant photosynthesis [14]. The second area of AMF action is the increased tolerance of the host plant to biotic and abiotic stressors, more effective nutrient and water absorption, higher photosynthetic activity, control of reactive oxygen species by increased activity of antioxidant enzymes [15–17]. AMF covers some basic (such as the alteration in root morphology, increased plant nutrition, and damage compensation) and secondary phenomena (competition between symbiotic and parasitic microorganisms, changes in rhizosphere microbial populations, and the activation of plant defense mechanisms) [18,19]. The phylum *Actinobacteria* represents a large group of non-mycorrhizal plant growth promoting microorganisms (PGPM), among them *Saccharothrix* spp. are aerobic, gram-positive actinomycetes with branching vegetative mycelium, that fragments into rod-shaped spores [20]. Merrouche et al. [21] have previously reported the intense activity of *Saccharothrix* against fungi (e.g., *Fusarium* spp.) and moderate activity against bacteria. PGPM and their positive impact on the plant have not been as broadly studied as AMF, although some studies have reported their beneficial effects on crops [22]. PGPM can shape the relationships in the rhizosphere microbiome through P solubilization, or induction of plant stress tolerance [23]. The significant beneficial effects of *Azospirillum* includes the capacity to fix atmospheric N, synthesize phytohormones and plant regulators, and increase plant tolerance to abiotic and biotic stresses [24]. Previous efforts to significantly change the indigenous microflora of the soil by introducing single cultures of extrinsic microorganisms have not always been successful. Thus, the probability of shifting the "microbiological equilibrium" of the rhizosphere and controlling it to favor the growth, yield, and health of crops is much greater if consortia of beneficial and effective microorganisms are introduced that are physiologically and ecologically compatible with one another [25–27]. When the consortia of AMF and PGPM become established, their individual beneficial effects are often magnified in a synergistic manner [28–30], although the relationships are not always simple. Clearly, the direct effects of PGPM on the trade-off among microbes and plants are still poorly understood regarding soil and crop fitness.

According to Pedersen et al. [31], agriculturally fit crops are defined as the most useful to humans in agricultural systems and the food industry. Tomato is a highly mycorrhizal species, and PGPM can act as stimulants for the growth and development of tomato plants directly or indirectly via the availability of many essential nutrients and phytohormones, and the suppression/destruction of plant diseases, decreasing oxidative stress, and activation of pathogenesis-related metabolites, as it had been reported for other crops [32–36]. Inui Kishi et al. [37] stated that PGPM can be successfully applied to manage crop fitness in degradable areas, because PGPM elicit so-called ‘induced systemic tolerance’ to salt, drought, and nutrient deficiency in rhizosphere, so tomatoes will benefit from PGPM when cultivated under easily degradable soil. The unresolved problem is that the PGPM-host relationships are highly dynamic and can evolve from symbiosis to parasitism when net costs of the symbiosis exceed net benefits [38]. Moreover, the symbionts enhance their own fitness, not necessarily the fitness of their host [32]. Thus, inoculation may result in a biotic stressor that triggers defense mechanisms in the plants. The analysis of plant physiological status and biochemical stress biomarkers can explain the nature of the reaction of the plant to inoculation under specific conditions of easily degradable soil. An innovative approach uses bacteria and fungi isolates and their consortia to directly link the effect to the microorganism species to understand the principles of the more complex biotrophic interactions [39]. The goal of the study was to evaluate the symbiotic microorganism effects on plant development under stressful conditions of soil susceptible to degradation. Specific goals include (i) evaluation of symbiotic microorganism effects on tomato seedling fitness under stressful conditions simulating those of soil susceptible to degradation; (ii) comparison of plant-microbial interaction after inoculation of microbial isolates and fungi-bacteria consortia; and (iii) development of an effective crop-microbial network, for the improvement of soil and plant status.

## Materials and methods

### Material and treatments

The experimental plant was the tomato *Solanum lycopersicum* L. cultivar Spencer F_1_ (Moravoseed Ltd., CZ). Tomato seedlings were cultivated in a growth chamber in pots filled with the different types of autoclaved (120 °C for 60 min) substrates. Three mixtures of sowing peat (Klasmann, DE) and sand (local source) were used, namely, peat:sand ratios of (v:v) (i) 100:0, (ii) 70:30, and (iii) 50:50. The pH was maintained at 6.5 by the use of calcium carbonate in relevant amounts. Selected substrate parameters are shown in Table 1. The substrates were inoculated with beneficial microorganisms: (i) arbuscular mycorrhizal fungi mix (AMF), (ii) AMF fungi and *Azospirillum brasilense* CCM 3862 (AMF + AZ), and (iii) *Saccharothrix tamanrassetensis* SA 198 (AMF + S). AMF mix contained *Claroideoglomus claroideum* BEG 96, *Claroideoglomus etunicatum* BEG 92, *Funneliformis geosporum* BEG 199, *Funneliformis mosseae* BEG 95, and *Rhizophagus irregularis* BEG 140, and was applied as an AMF mixture containing 145 spores per g at a dose of 0.015 g cm^3^ of the substrate. The AMF + AZ treatment contained AMF and *A*. *brasilense* (10^8^ CFU in sterile 1XPBS). Seeds were soaked for 30 min. and were used for tomato inoculation with *A*. *brasilense*. *S*. *tamanrassetensis* was grown in a yeast-malt extract liquid medium (ISP2) from the International Streptomyces Project [40] at 28 °C with agitation at 90 rpm for 10 d. Next, the culture was homogenized using sterile ceramic beads and the suspension was adjusted to 0.5 OD600 (concentration measured at 600 nm) in sterile physiological saline. The control treatment (C) was a substrate without inoculation with AMF or bacteria.

**Table 1:** Selected substrate parameters at the beginning of the experiment.

Peat: sand treat-ment	pH	Dry matter	N-NO_3_	N-NH_4_	N total	K	P	Mg	Na	Ca	Cation exchange capacity	Soil weight per pot	Substrate bulk density
	CaCl	%	mg kg^–1^	mg kg^–1^	mg kg^–1^	mg kg^–1^	mg kg^–1^	mg kg^–1^	mg kg^–1^	mg kg^–1^	mM kg^–1^	g	kg m^–3^
50/50	6.53	93.0	5.2	1.9	7.0	505	61	596	92	4435	284	386	755
70/30	6.64	90.2	6.7	2.3	8.9	684	81	785	113	4741	319	311	606
100/0	6.47	86.1	8.9	2.9	11.8	953	111	1070	144	5200	372	163	259

#### Cultivation conditions

Seeds were sterilized for 10 min in 3% sodium hypochlorite and washed with sterile distilled water. Sowing was conducted on April 2, 2020 directly into Teku V9 containers (square shape 9 × 9 cm, height 8 cm, volume 512 cm^3^). Cultivation was performed in phytochamber Fytoscope 4400 (PSI, CZ) with the temperature at the germination stage of 23/20 °C (day/night). At 5 d, the cotyledons stage began and the temperature was reduced to 20/18 °C, followed by 22/19 °C. Relative air humidity was maintained at 85% for germination, followed 75% for the subsequent experimental period. Light intensity was set to 140 μmol m^-2^ s^-1^ for the germination stage, then increased to 200 μmol m^-2^ s^-1^ for the subsequent period of the trial, with 16 h of daylight. Irrigation with tap water was applied equally to all pots at a measured volume. Urea was used for fertilization (May 5, liquid 0.2% solution, 20 cm^3^ per pot in irrigation doses), later the fertilizer YaraTera Kristalon 20 + 5 + 10 + 2 Azur was applied May 13 and 27 as a 0.1% liquid solution at a dose of 20 cm^3^ per pot.

#### Substrate sampling and analyses

After the end of the experiment (June 3, 2020), samples of the substrate were taken to conduct laboratory analyses. A sample of approximately 150 g was taken from each replicate. The collected samples were air-dried, and basic parameters were determined, including pH (H_2_O and KCl) using the potentiometric method, level of salinity using the conductometric method, and the capacity of the sorption complex by Kappen method. The content of total N and organic C was determined by elemental analysis using the Vario Max Cube apparatus (Elementar Analysensysteme GmbH, Langenselbold, Germany). Available forms of macroelements (Ca, Mg, Na, S, K, P) and microelements (Mn, Fe, Zn, Cu) were determined in the substrate samples by means of acetic acid after prior extraction according to the Nowosielski method [41]. The elements were determined by inductively coupled plasma atomic emission spectrometry using a Perkin Elmer Optima 7600 (PerkinElmer, US) spectrometer.

#### Plant material sampling

Tomato samples were collected on June 3, 2020, when all leaves were cut with scissors. Roots were completely extracted from the substrate and washed with distilled H_2_O. Samples were stored immediately after harvest in a -80 °C deep freezer until analyses. For colonization analysis, root subsampling was conducted by selecting 10–20 mm randomly selected roots per container. These were fixed in a formaldehyde:ethanol:acetic acid 10%:50%:5% v/v solution (FAA) and stored in the dark at 4 °C before staining for microscopy [42].

Total aboveground fresh leaf weight and root fresh weight (FW) per plant was measured in five replicates per treatment.

#### Staining and microscopy

After fixation in FAA, roots were rinsed in distilled H_2_O, then cleared in 2% KOH solution (1 h at 50 °C), and washed in distilled H_2_O (4 × 3 min). Roots were stained with a 0.03% (w/v) solution of Uvitex2B for 45 min at 90 °C, rinsed in distilled H_2_O, and incubated in H_2_O for 12 h. When the roots were placed on slides, a few drops of Hoechst/DAPI were added and covered with cover slip [43]. For staining with Alexa Fluor (AF) conjugates of wheat germ agglutinin (WGA), concanavalin A (Con A), and acid fuchsine, the tissues were fixed and cleared using the method described above. Roots were stained in a tube with a mixture consisting of WGA AF 594 conjugate (InvitrogenTM, USA) (50 μg mL^–1^), Con A AF 647 (InvitrogenTM, USA) (50 μg mL^–1^), and acid fuchsine (3%) at a ratio of 1:1:1, for 4–5 h at room temperature, rinsed in PBS (4 × 3 min) and incubated for 12 h in PBS to remove all excess stain. Before mounting on the slide, few drops of Hoechst stain were added to the slides with roots [44]. Mycorrhizal colonization quantification was conducted according to the grid-intersect method [45].

Confocal microscopy was completed using the LSM 800 (Carl Zeiss, Germany) microscope at 405/420–480 and 488/500–550 nm excitation/emission for Uvitex2B, 590/617 nm (excitation/emission) for WGA AF 594, 650/668 nm for Con A AF 647, and 350/461 nm for Hoechst stain. Lens used was 20x/0.8 NA. Processing of pictures was conducted in Zen Blue 3.0 (Carl Zeiss, Germany).

#### Physiological parameters

Plants at the stage of 30–40 cm height and having at least eight developed leaves were evaluated. Analysis of Normalized Difference Vegetation Index (NDVI) was performed on tomatoes before leaf harvest on May 25 by PlantPen model NDVI 310 (PSI, Ltd, CZ), a 50 mm^2^ detector measuring at 635 and 750 nm. Seven fully developed leaves (the fourth from the plant apex) were analyzed per treatment. Chlorophyll absorbs visible light from 0.4 to 0.7 μm for photosynthesis, and cell structures strongly reflect near-infrared light from 0.7 to 1.1 μm. The differences in reflectance in the visible and near-infrared wavelengths were used to calculate the NDVI index.

On the same date (May 25), Quantum Yield (QY) was analyzed by the FluorPen model FP 110 (PSI Ltd, CZ) using seven tomato leaves per treatment (the same leaves as used for NDVI, but different leaf blade position). QY reflects photosystem II efficiency. In a 20 min dark-adapted leaf, this is equivalent to Fv/Fm.

#### Analyses of stress biomarkers

The antioxidant activity was determined in plant samples following DPPH radical (2,2-diphenyl-1-picrylhydrazyl) scavenging method [46]). The absorbance at λ = 517 nm of the mixture containing 0.1 cm^3^ supernatant and 4.9 cm^3^ 0.1 mM DPPH in 80% methanol was measured after 15 min of incubation in darkness at 20–22 °C with the UV-VIS Helios Beta spectrophotometer (Thermo Fisher Scientific, Inc., US). The antioxidant activity was calculated with the following formula: DPPH (%) = ((A0 − A1)/A0) × 100; where A0 is the absorbance of the reference solution and A1 is the absorbance of the test solution [47].

The total phenolics was estimated using the modified Folin-Ciocalteu colorimetric method [48]. A 2.5 g sample of leaves was mixed with 10 cm^3^ of 80% methanol and centrifuged (3 492 × *g*, 15 min, 4 °C). The glass tubes were filled with 0.1 cm^3^ of the supernatant and 2 cm^3^ of sodium carbonate, left for 5 min, and then 0.1 cm^3^ of Folin-Ciocalteu’s reagent, mixed with deionized water (1:1 v/v) was added. After 45 min, phenols were determined by the colorimetric method at 750 nm using UV-VIS spectrophotometer against a reference solution. The total phenol value was expressed as gallic acid equivalents (mg GAE) per g FW.

To determine glutathione peroxidase (GPOX) activity, 2.5 g of leaves was grounded in an ice bath with 20 cm^3^ of a 0.05 M potassium phosphate buffer and centrifuged (3 492 × *g*, 15 min, 4 °C). Then GPOX was assayed, according to Lück [49], with p-phenylenediamine as an electron donor and hydrogen peroxide as an oxidant. The reaction mixture contained the diluted supernatant, 0.05 M potassium phosphate buffer, p-phenylenediamine, and hydrogen peroxide. The absorbance at 485 nm was recorded at 60 s intervals for 2 min using a UV-VIS spectrophotometer. The GPOX activity was expressed in units (U) per g FW per min.

#### Element concentration in plant tissues

To determine the micro- and macroelement concentrations in the dry tomato samples, roots and leaves were mineralized in a mixture of HNO_3_ solution and H_2_O_2_ at 1:3 (v:v). The weight of an analytical sample was max. 0.5 g dry weight (DW). Samples were acidified by adding 2 cm^3^ HNO_3_ per 100 cm^3^ distilled water. The samples were concentrated 5-fold, and then the concentrations of elements (Ca, Mg, Na, S, K, P, Mn, Fe, Zn, and Cu) in the prepared solutions were determined by atomic emission spectroscopy (ICP) with an Optima 7600 spectrophotometer (Perkin Elmer, US), using the method described by Pasławski and Migaszewski [50].

#### Statistical analysis

The experiment was designed in a randomized complete block with seven replicates and two factors, representing different substrate compositions and different inocula used and the non-inoculated control. The substrate and plant samples for stress biomarkers and element concentrations were taken separately from every treatment and determined using three technical replicates. The samples for physiological parameters were measured using seven biological replications. The data were tested for normality of distribution according to the Shapiro and Wilk method [51] and homogeneity of variances using the Levene test [52]. A one- or two-way ANOVA was applied to test significance levels at *P ≤* 0.05 (*), *P ≤* 0.01 (**), or *P* ≤ 0.001 (***) and non-significance (ns). Tukey’s HSD (honest significant difference) test was used to separate means into homogenous groups. In the case of the one-way ANOVA, the experimental treatments were the source of variation. In the case of two-way ANOVA—plant parts (leaves and roots) were additional source of variation. The results were also examined using Pearson’s correlation coefficient (r) between analyzed parameters. The principal component analysis (PCA), cluster (CA) analysis and heat maps were performed to precisely demonstrate and analyze the data and relationships among them. Correlations and PCA were used as supplementary statistical methods that enabled and expanded the analysis of the presented data and made additional relationships visible between experimental treatments and parameters. The results using raw data were presented for PCA analysis because no substantial differences appeared between raw and standardized data. All analyses were performed with the software Statistica 13.0 (Dell, Inc., USA).

## Results

### Substrate properties at the end of the experiment

The differences in the values characterizing the sorption complex in the substrate resulted from the share of the organic fraction in the substrates used in the experiment. The organic carbon content in the treatments C 100, AMF + AZ 100, and AMF + S 100 was approximately 50%, whereas, in the substrates of the remaining, this value ranged from approximately 3 to 7% of the DW of the substrate (Table 2). A statistically significant negative correlation occurred between the capacity of the sorption complex and the pH of the substrate (r = -0.985, *P ≤* 0.01) and between the capacity of the sorption complex and the content of organic carbon (r = -0.969, *P ≤* 0.01). A statistically significant positive correlation coefficient was found between the capacity of the sorption complex and salinity (r = 0.675, *P ≤* 0.05). In the case of the relationship between pH and salinity, the value of the correlation coefficient was r = -0.686, *P ≤* 0.05. The research results indicated different amounts of trace element forms available for plants in the substrates or treatment.

**Table 2:** Effects of soil microorganisms on substrate physical and chemical characteristics after tomato transplants cultivation.

Treatment	Sum in the sorption complex (mM Na^+^ kg^–1^)		S + H (mM kg^–1^)	Cation exchange capacity with alkaline cations (%)	Organic carbon (%)	Total nitrogen (%)	EC (mS)	pH	
	alkaline cations (S)	acid cations (H)						H_2_O	HCl
C 50*	320	44	364	87.9	2.94	0.312	0.32	7.48	7.01
C 70	512	56	568	90.1	6.82	0.649	1.45	7.08	6.70
C 100	1352	172	1524	88.7	51.77	4.869	1.36	6.27	5.92
AMF + AZ 50	396	52	448	88.4	2.91	0.303	0.99	7.06	6.86
AMF + AZ 70	516	48	564	91.5	5.90	0.612	1.37	7.05	6.76
AMF + AZ 100	1296	144	1440	90.0	49.18	4.682	1.48	6.55	6.17
AMF + S 100	1272	148	1420	89.6	50.72	4.882	2.06	6.50	6.15

*C 50 –peat:sand ratio 50:50 (v:v) without inoculation; C 70 –peat:sand ratio 70:30 (v:v) without inoculation; C 100 –peat:sand ratio 100:0 (v:v) without inoculation; AMF + AZ 50 –peat:sand ratio 50:50 (v:v) inoculated with arbuscular mycorrhizal fungi (AMF) and Azospirillum brasilense (AZ), AMF + AZ 70 –peat: sand ratio 70:30 (v:v) inoculated with AMF and AZ, AMF + AZ 100 –peat:sand ratio 100:0 (v:v) inoculated with AMF and AZ; AMF + S– 100 peat:sand ratio 100:0 (v:v) inoculated with AMF and Saccharothrix tamanrassetensis (S).

The greatest differentiation in the content of available forms of elements for plants was found for sodium (Na), phosphorus (P), and sulfur (S). The relative standard deviation for these elements was approximately 90%. The smallest variation occurred for calcium (Ca) and iron (Fe), where the variability in individual treatments was approximately 20%. The content of Ca and potassium (K) forms available to plants was similar, regardless of treatment. Potassium content in substrates after plant cultivation was in a range of 21 to 147 mg kg^-1^ DW. In contrast, substrates composed of peat, with the addition of beneficial microorganisms (AMF + AZ 100 and AMF + S 100) contained the highest level of K after cultivation, and those composed of peat:sand in the ratio of 50:50 (v:v) contained the lowest (C 50, AMF + AZ 50) (Table 3). Calcium content in the substrate after tomato cultivation was the lowest in the control treatment composed of peat:sand at the ratio of 70:30 (v:v) (C 70), whereas it was significantly higher in the C 50 treatment and all treatments composed of peat (C 100, AMF + AZ 100, and AMF + S 100). In general, treatments composed of peat:sand in the ratio of 50:50 (v:v) and 70:30 (v:v), despite microorganism application, contained the lowest amounts of soluble S, K, P, magnesium (Mg), zinc (Zn), and copper (Cu) after tomato cultivation, which was opposite to that of substrates composed of peat (C 100, AMF + AZ 100, and AMF + S 100) (Table 3). The highest Cu contents were found in the C 100, AMF 100, and AMF + S 100 treatments. The content of this element in the substrates of these treatments was approximately 6-times higher than in the C 50 and AMF + AZ 50 treatments. A similar relationship was found in the case of S and P. The substrate composed with peat:sand in the ratio of 50:50 (v:v) contained the highest amount of soluble manganese (Mn), whereas that formulated with peat enriched with beneficial microorganisms consortium (AMF + S 100) was characterized by the lowest Mn content after tomato cultivation. In the case of most of the examined elements, a statistically significant correlation occurred between the content of their assimilable forms in the media. The most common was a negative correlation between the reaction and the content of elements in the media and a positive correlation between organic C and the amount of assimilable forms of elements. The exceptions were Fe and Mn, where the relationships were opposite. The values of the correlation coefficients of most elements were approximately 0.9 (Table 4).

**Table 3:** Effects of soil microorganisms on soluble minerals (mg kg^-1^ DW) in substrate after tomato transplants cultivation.

**Treatment**	**Ca**	**Mg**	**Na**	**S**	**K**
C 50*	2619 ± 114 bc	273 ± 11 a	230 ± 14 a	205 ± 15 a	21 ± 0.8 a
C 70	1979 ± 51 a	299 ± 12 a	271 ± 15 a	232 ± 55 a	33 ± 7.1 a
C 100	3105 ± 170 d	972 ± 62 c	1614 ± 179 c	1687 ± 168 c	86 ± 5.4 bc
AMF + AZ 50	2362 ± 70 a-c	255 ± 21 a	206 ± 16 a	209 ± 53 a	33 ± 4.1 a
AMF + AZ 70	2272 ± 127 ab	384 ± 28 a	317 ± 26 a	318 ± 22 a	52 ± 4.0 ab
AMF + AZ 100	2768 ± 298 c	865 ± 145 bc	1159 ± 329 b	1684 ± 40 c	147 ± 35.0 d
AMF + S 100	2526 ± 92 bc	740 ± 35 b	852 ± 113 b	1143 ± 116 b	104 ± 10.3 c
	**P**	**Mn**	**Fe**	**Zn**	**Cu**
C 50	9.35 ± 0.4 a	11.5 ± 0.18 d	2.57 ± 0.17 b	0.56 ± 0.01 a	0.054 ± 0.01 a
C 70	15.63 ± 3.7 a	8.42 ± 0.02 c	2.48 ± 0.19 b	0.51 ± 0.00 a	0.087 ± 0.00 a
C 100	86.30 ± 12.8 c	5.58 ± 0.23 b	1.66 ± 0.02 a	1.14 ± 0.06 b	0.320 ± 0.03 c
AMF + AZ 50	11.42 ± 1.45 a	8.51 ± 0.04 c	1.74 ± 0.18 a	0.57 ± 0.09 a	0.046 ± 0.00 a
AMF + AZ 70	16.93 ± 0.4 a	8.94 ± 0.19 c	2.30 ± 0.03 b	0.58 ± 0.05 a	0.100 ± 0.01 a
AMF + AZ 100	70.19 ± 8.2 c	5.03 ± 0.55 b	2.27 ± 0.26 b	0.95 ± 0.15 b	0.291 ± 0.05 bc
AMF + S 100	46.69 ± 3.9 b	4.31 ± 0.04 a	1.74 ± 0.06 a	0.90 ± 0.14 b	0.247 ± 0.02 b

*C 50 –peat:sand ratio 50:50 (v:v) without inoculation; C 70 –peat:sand ratio 70:30 (v:v) without inoculation; C 100 –peat:sand ratio 100:0 (v:v) without inoculation; AMF + AZ 50 –peat:sand ratio 50:50 (v:v) inoculated with arbuscular mycorrhizal fungi (AMF) and Azospirillum brasilense (AZ), AMF + AZ 70 –peat:sand ratio 70:30 (v:v) inoculated with AMF and AZ, AMF + AZ 100 –peat:sand ratio 100:0 (v:v) inoculated with AMF and AZ; AMF + S– 100 peat:sand ratio 100:0 (v:v) inoculated with AMF and Saccharothrix tamanrassetensis (S).

**Table 4:** The value of the correlation coefficients between the individual substrate parameters after the end of the experiment.

	Ca	Mg	Na	S	K	P	Mn	Fe	Zn	Cu
pH	−0.656	−0.972**	−0.950**	−0.950**	−0.832**	−0.956**	0.923**	0.583	−0.953**	−0.980**
Corg	0.702*	0.972**	0.922**	0.964**	0.893**	0.933**	−0.915**	−0.530	0.952**	0.979**

** Correlation coefficient significant at P ≤ 0.01.

### Plant fresh weight

**Fig 1:**
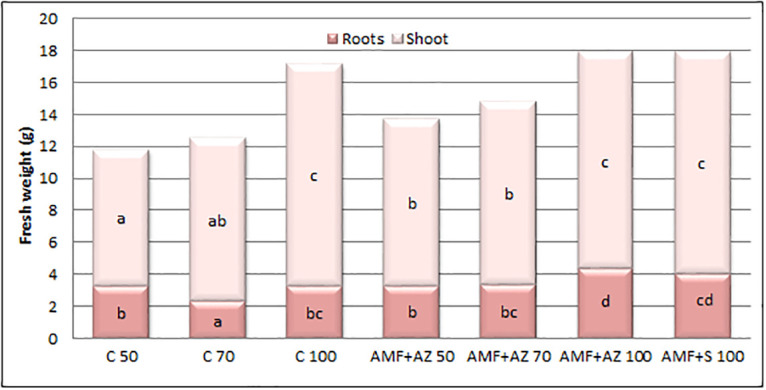
Effects of soil microorganisms application on fresh weight of tomato shoots and leaves. Means followed by different letters are significantly different at *p ≤* 0.05, with comparisons performed by Fisher’s LSD test, separately for roots and shoots. C 50 –peat:sand ratio 50:50 (v:v) without inoculation; C 70 –peat:sand ratio 70:30 (v:v) without inoculation; C 100 ––peat:sand ratio 100:0 (v:v) without inoculation; AMF + AZ 50 –peat:sand ratio 50:50 (v:v) inoculated with arbuscular mycorrhizal fungi (AMF) and *Azospirillum brasilense* (AZ), AMF + AZ 70 ––peat:sand ratio 70:30 (v:v) inoculated with AMF and AZ, AMF + AZ 100 –peat:sand ratio 100:0 (v:v) inoculated with AMF and AZ; AMF + S–– 100 peat:sand ratio 100:0 (v:v) inoculated with AMF and *Saccharothrix tamanrassetensis* (S).

Fresh weight of leaves (mean 11.66 g) and roots (mean 3.44 g) was evaluated after extraction of samples from containers and exhibited significant treatment effects dependent upon the differences in the parameters (Fig 1). Although the lowest leaf and root FW values were recorded at C 50 and C 70, respectively, the highest leaf FW occurred in all treatments with peat media only (AMF + AZ 100, AMF + S 100, and C 100) showing the significantly positive impact of peat on plant growth. Treatment AMF + AZ 100 exhibited a significantly high root FW, together with AMF + S 100. The ratio of shoot/root weight was recorded in the range of 3.10 (C 70) to 3.95 (AMF + AZ 100) and leaf weight was correlated with root weight (r = 0.4781).

### Root colonization

**Fig 2:**
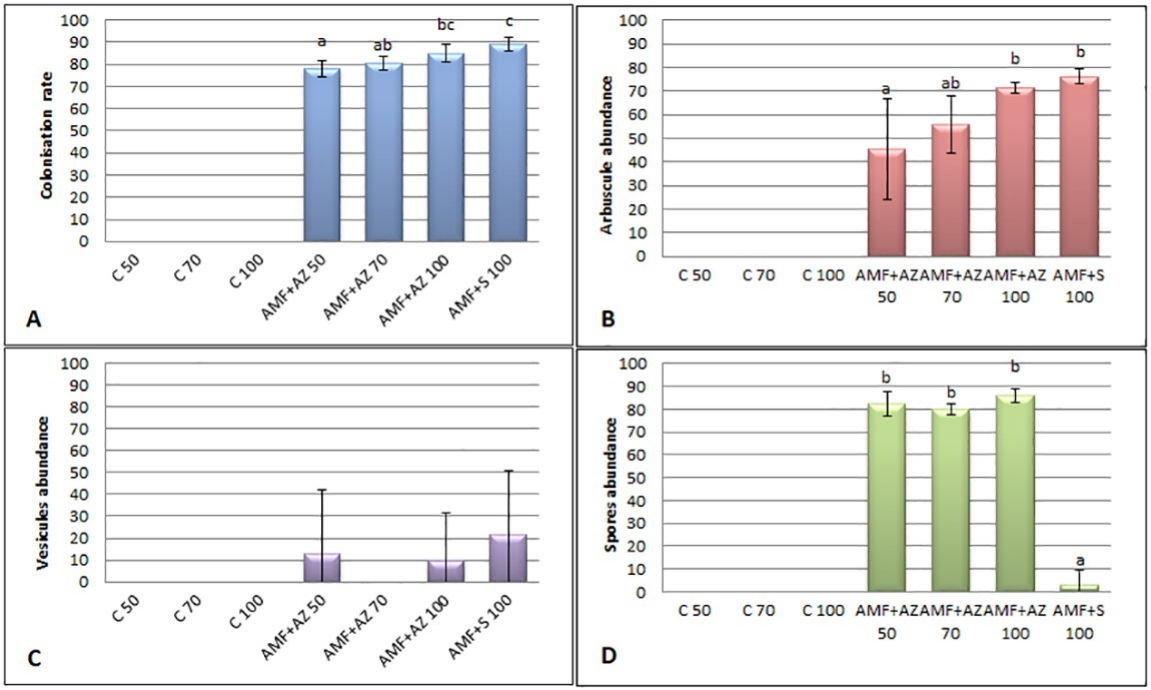
Effects of soil microorganisms application mycorrhization parameters of tomato roots: Colonization rate (A), arbuscule abundance (B), vesicules abundance (C), and spores abundance (D). Means followed by different letters are significantly different at *P ≤* 0.05, with comparisons performed by Fisher’s LSD test, only for mycorrhized objects. C 50—peat:sand ratio 50:50 (v:v) without inoculation; C 70 –peat:sand ratio 70:30 (v:v) without inoculation; C 100 –peat:sand ratio 100:0 (v:v) without inoculation; AMF +AZ 50 –peat:sand ratio 50:50 (v:v) inoculated with arbuscular mycorrhizal fungi (AMF) and *Azospirillum brasilense* (AZ), AMF + AZ 70 –peat:sand ratio 70:30 (v:v) inoculated with AMF and AZ, AMF + AZ 100 –peat:sand ratio 100:0 (v:v) inoculated with AMF and AZ; AMF + S– 100 peat:sand ratio 100:0 (v:v) inoculated with AMF and *Saccharothrix tamanrassetensis* (S).

Results of confocal microscopy confirmed successful root colonization of mycorrhizal fungi in all treatments inoculated by AMF. Data in Fig 2A show the high mean level for root colonization rate in AMF + AZ 50, AMF + AZ 70, AMF + AZ 100, and AMF + S 100 in the range from 78 to 89%. Root colonization in the control treatment was not detected.

Following root colonization rate, arbuscular abundance was analyzed. As Fig 2B shows, the arbuscules were present from 45 to 76%. These levels indicated the intensive process of mutual symbiosis. Mycorrhizal fungi also developed spores detected by microscopic observation (Fig 2D).

Their presence occurred in AMF + AZ 50, AMF + AZ 70, and AMF + AZ 100, but not in AMF + S.

Supporting figures from microscopic observation demonstrate results. S1 and S2 Figs show the interaction of mycorrhizal fungi in root tissue and bacterial colonies in the root hair area. Root samples carefully extracted from the substrate revealed the development of dense mycelial structures surrounding root hairs of tomatoes (S1_1 Fig). Conditions with higher organic matter content led to more abundant grid structures of fungi on roots. In most of the AMF treatments, the development of spores was detected. This situation was also found in the low organic matter content treatment (AMF + AZ 50) and confirmed the ability of AMF to colonize plant roots and form propagative structures (S1_2 Fig).

S1_3A and S1_3B Fig show effective symbiosis between mycorrhizal fungi and tomato plants. Arbuscular structures in root tissues correspond to positive interactions of host and fungi in water/nutrient exchange. Treatments with 100% peat showed higher levels of arbuscules abundance, although symbiosis functioned in the peat:sand substrate (50:50) as well.

Treatments containing the bacterial inoculant *Azospirillum brasilense* were observed for the determination of bacterial colonies on roots. In all treatments with this inoculation, the bacterial colonies were abundant, as shown by the specific probe for *A*. *brasilense* (S2 and S3 Figs).

### Physiological parameters

**Fig 3:**
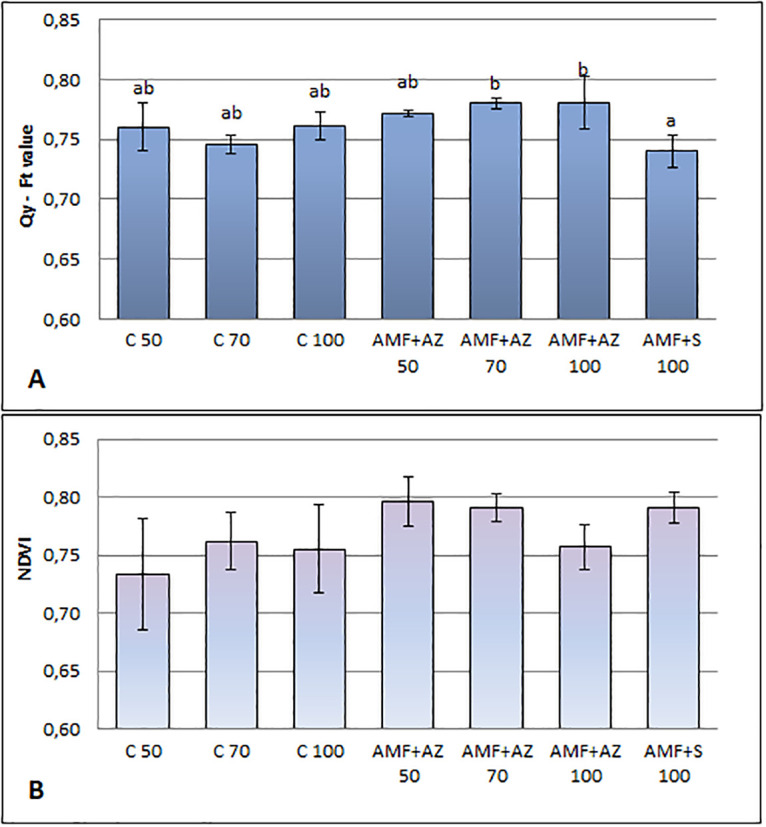
Effects of soil microorganisms application to substrates of different organic matter content on Quantum Yield (QY) (A) and Normalized Difference Vegetation Index (NDVI) (B) in tomato leaves transplants. Means followed by different letters are significantly different at *P ≤* 0.05, with comparisons performed by Fisher’s LSD test. Error bars represent the standard deviation (± SD) for interaction. C 50 –peat:sand ratio 50:50 (v:v) without inoculation; C 70 –peat:sand ratio 70:30 (v:v) without inoculation; C 100 –peat:sand ratio 100:0 (v:v) without inoculation; AMF + AZ 50 –peat:sand ratio 50:50 (v:v) inoculated with arbuscular mycorrhizal fungi (AMF) and *Azospirillum brasilense* (AZ), AMF + AZ 70 –peat:sand ratio 70:30 (v:v) inoculated with AMF and AZ, AMF + AZ 100 –peat:sand ratio 100:0 (v:v) inoculated with AMF and AZ; AMF + S– 100 peat:sand ratio 100:0 (v:v) inoculated with AMF and *Saccharothrix tamanrassetensis* (S).

The NDVI as a parameter corresponding to the physiological status of plants was 0.77 (mean for all treatments), according to the evaluation of tomato leaf fluorescence activity. The higher NDVI levels occurred in AMF + AZ 50,70 and AMF + S. The NDVI of control treatments was lower, especially in C 50, but differences were not significant statistically (Fig 3).

**Fig 4:**
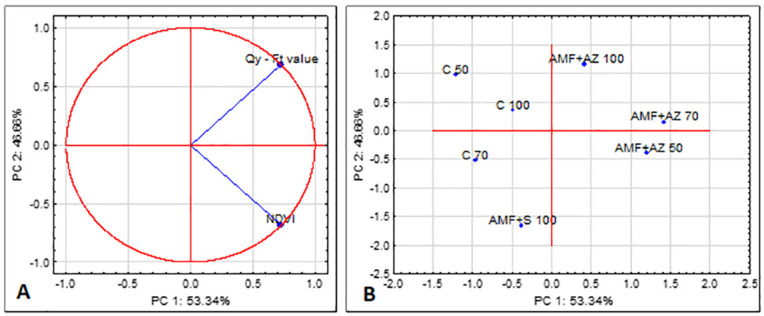
Bi-plot presenting the correlation between the tested fluorescence parameters of tomato transplant leaves (A) and ordination illustrating differences between substrates tested towards the fluorescence parameters of tomato transplant leaves (B). C 50 –peat:sand ratio 50:50 (v:v) without inoculation; C 70 –peat:sand ratio 70:30 (v:v) without inoculation; C 100 –peat:sand ratio 100:0 (v:v) without inoculation; AMF + AZ 50 –peat:sand ratio 50:50 (v:v) inoculated with arbuscular mycorrhizal fungi (AMF) and *Azospirillum brasilense* (AZ), AMF + AZ 70 –peat:sand ratio 70:30 (v:v) inoculated with AMF and AZ, AMF + AZ 100 –peat:sand ratio 100:0 (v:v) inoculated with AMF and AZ; AMF + S– 100 peat:sand ratio 100:0 (v:v) inoculated with AMF and *Saccharothrix tamanrassetensis* (S).

Quantum yield expressed by the Ft value exhibited a non-significant correlation with NDVI, where Qy = 0.76718–0.0055*NDVI corresponding to the tight link among both physiological parameters (Fig 4). There was no significant difference in this value among control treatments. The values ranged from 70 AMF + AZ and 100 to the AMF + S with the lowest value.

### Analyses of stress biomarkers

**Fig 5:**
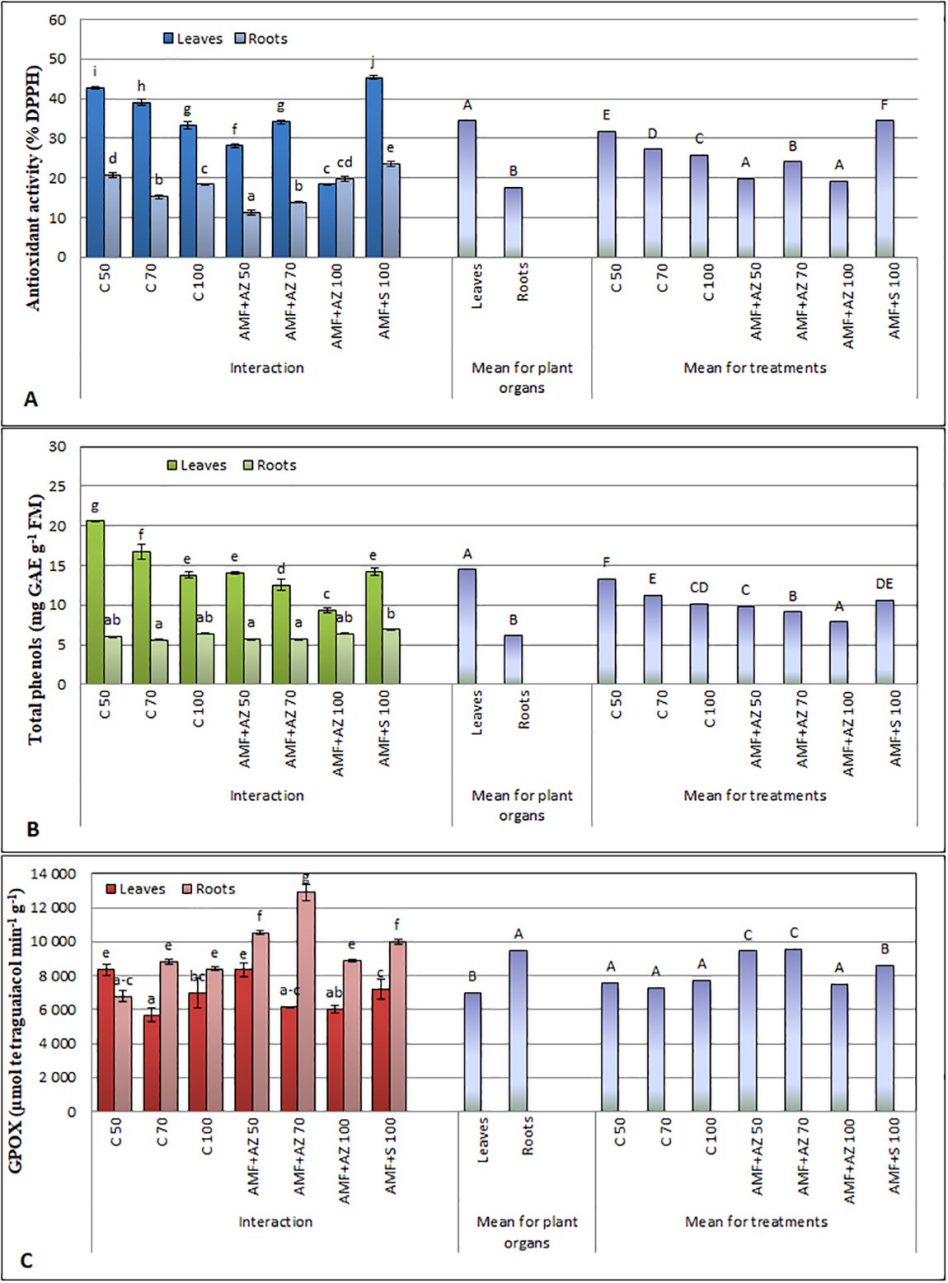
Effects of soil microorganisms application to substrates of different organic matter content on antioxidant activity (A), total phenols (B), and glutathione peroxidase (GPOX) (C) activity in leaves and roots of tomato transplants. Means followed by different letters are significantly different at *P ≤* 0.05, with comparisons performed by Fisher’s LSD test. Error bars represent the standard deviation (± SD) for interaction. C 50 –peat:sand ratio 50:50 (v:v) without inoculation; C 70 –peat:sand ratio 70:30 (v:v) without inoculation; C 100 –peat:sand ratio 100:0 (v:v) without inoculation; AMF + AZ 50 –peat:sand ratio 50:50 (v:v) inoculated with arbuscular mycorrhizal fungi (AMF) and *Azospirillum brasilense* (AZ), AMF + AZ 70 –peat: sand ratio 70:30 (v:v) inoculated with AMF and AZ, AMF + AZ 100 –peat:sand ratio 100:0 (v:v) inoculated with AMF and AZ; AMF + S– 100 peat:sand ratio 100:0 (v:v) inoculated with AMF and *Saccharothrix tamanrassetensis* (S).

**Fig 6:**
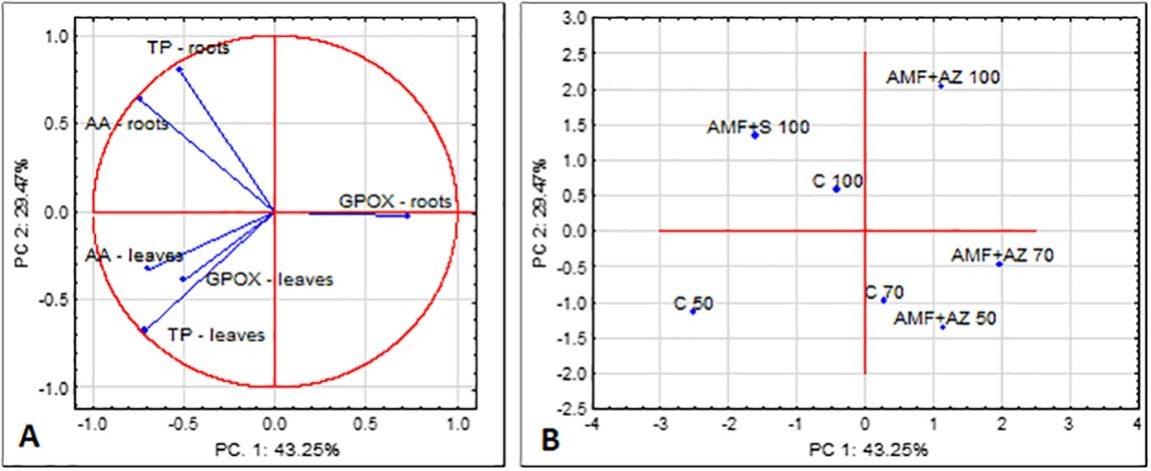
Bi-plot presenting the correlation between the tested antioxidant parameters of tomato transplants (A) and ordination illustrating differences between substrates tested towards the antioxidant parameters of tomato transplants (B). C 50 –peat:sand ratio 50:50 (v:v) without inoculation; C 70 –peat:sand ratio 70:30 (v:v) without inoculation; C 100 –peat:sand ratio 100:0 (v:v) without inoculation; AMF + AZ 50 –peat:sand ratio 50:50 (v:v) inoculated with arbuscular mycorrhizal fungi (AMF) and *Azospirillum brasilense* (AZ), AMF + AZ 70 –peat:sand ratio 70:30 (v:v) inoculated with AMF and AZ, AMF + AZ 100 –peat:sand ratio 100:0 (v:v) inoculated with AMF and AZ; AMF + S– 100 peat:sand ratio 100:0 (v:v) inoculated with AMF and *Saccharothrix tamanrassetensis* (S).

The antioxidant activity was considered in roots and shoots of tomato seedlings affected by different growth conditions followed by physiological acclimatization processes. Generally, the antioxidant activity measured as DPPH scavenging activity was approximately 2-times higher in tomato leaves than in roots for each treatment, except AMF + AZ 100 (Fig 5A). In general, AM fungi and *Azospirillum brasilense* (AMF + AZ) applied to the substrate reduced tomato seedling antioxidant activity. Tomato seedlings, grown in the substrate composed of peat and inoculated with AMF and *Saccharothrix tamanrassetensis* (AMF + S), showed the highest antioxidant activity. The roots of plants cultivated in a substrate composed of peat:sand at the ratio 50:50 (v:v) and inoculated with AMF + AZ. The lowest DPPH scavenging activity in roots was recorded in treatment AMF + AZ in substrate with 50:50 peat:sand ratio, while the highest DPPH activity in the leaves at AMF + S treatment was found. Antioxidant activity of tomato leaf extracts was positively correlated with total phenolics in leaves (Table 1), a similar relation was observed for roots. These dependences are illustrated (Fig 6A) by the distance of the eigenvectors of antioxidant activity and total phenolics and narrow angles between the eigenvectors.

The total phenolics (TP) content was significantly higher in tomato seedling leaves than roots, considering the main effects analysis (Fig 5B). Tomato roots contained a similar phenolics level, and significant differences were noted for C 70, AMF + AZ 50, and AMF + AZ 70 treatments, whereas the AMF + S 100 treatment had a higher level. Analysis of the TP in tomato leaves shoved its higher level in samples from plants of C 50 and C 70 treatments, without microbiota inoculation. Total phenolics in tomato leaves were positively correlated with Ca Mg, Na, and Mn contents in leaves and roots, Fe in roots, and Cu in leaves. The correlations of TP and mineral elements in tomato roots were positive for S and Zn, whereas a negative relationship was noted for Ca, Mn, and Fe. The correlations of TP in tomato roots and mineral elements in tomato leaves were positive only for Fe, but negative for K, Ca, Mg, Na, and Mn (Table 2).

The tomato seedling roots showed a higher GPOX activity than leaves, except for the plants grown in peat:sand at a ratio of 50:50 (v:v), without microbiota inoculation (Fig 5C). The most notable differences were for roots (the lowest GPOX activity) and the roots (the highest GPOX activity) of tomato seedlings sampled from the substrate with peat:sand ratio 70:30 (v:v), inoculated with AMF + AZ. GPOX activity in roots was 2-times higher than in leaves of the seedlings of this treatment. The conversely directed eigenvectors of GPOX activity and total phenolics in roots of tomato seedlings in Fig 4a illustrated the negative correlation between these parameters, with a correlation coefficient of r = -0.514, *P ≤* 0.05. However, the correlation between the aforementioned parameters in tomato leaves was positive. Correlation coefficients between GPOX activity of tomato seedlings roots and shoots are presented in Table 2. PCA analysis illustrated that the C 50 treatment contributed significantly and negatively to PC1 and PC2, whereas AMF + AZ 100 contributed positively to PC1 and PC2 (Fig 6B).

### Elements concentration in plant tissues

**Fig 7:**
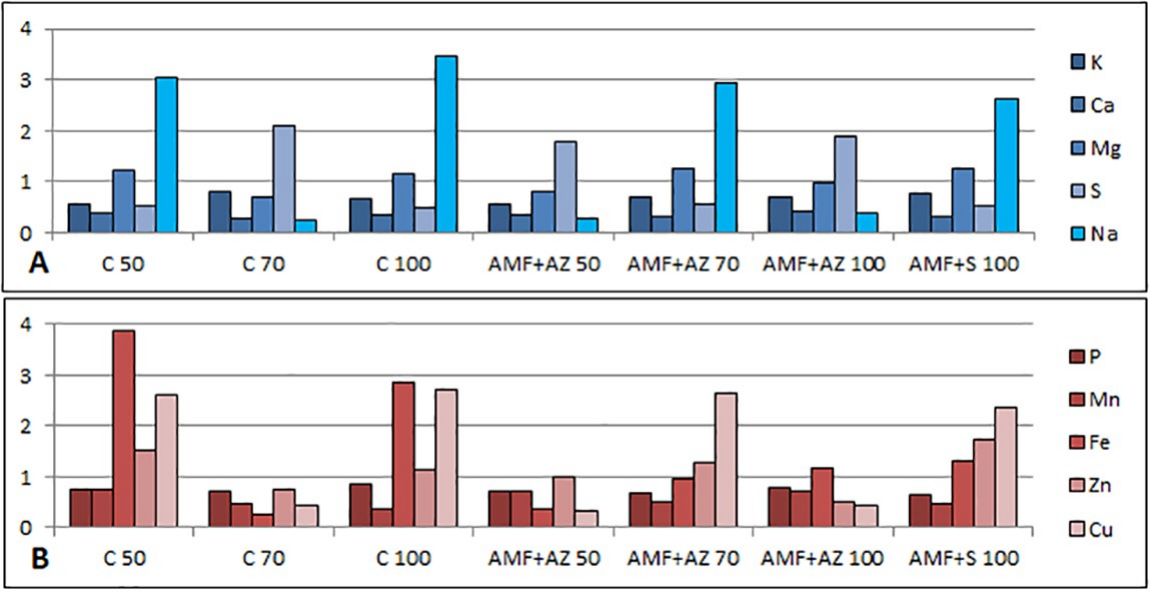
Effects of soil microorganisms application to substrates of different organic matter content on root: Shoot ratio of mineral elements in tomato transplants. C 50 –peat:sand ratio 50:50 (v:v) without inoculation; C 70 –peat:sand ratio 70:30 (v:v) without inoculation; C 100 –peat:sand ratio 100:0 (v:v) without inoculation; AMF + AZ 50 –peat:sand ratio 50:50 (v:v) inoculated with arbuscular mycorrhizal fungi (AMF) and *Azospirillum brasilense* (AZ), AMF + AZ 70 –peat:sand ratio 70:30 (v:v) inoculated with AMF and AZ, AMF + AZ 100 –peat:sand ratio 100:0 (v:v) inoculated with AMF and AZ; AMF + S– 100 peat:sand ratio 100:0 (v:v) inoculated with AMF and *Saccharothrix tamanrassetensis* (S).

**Fig 8:**
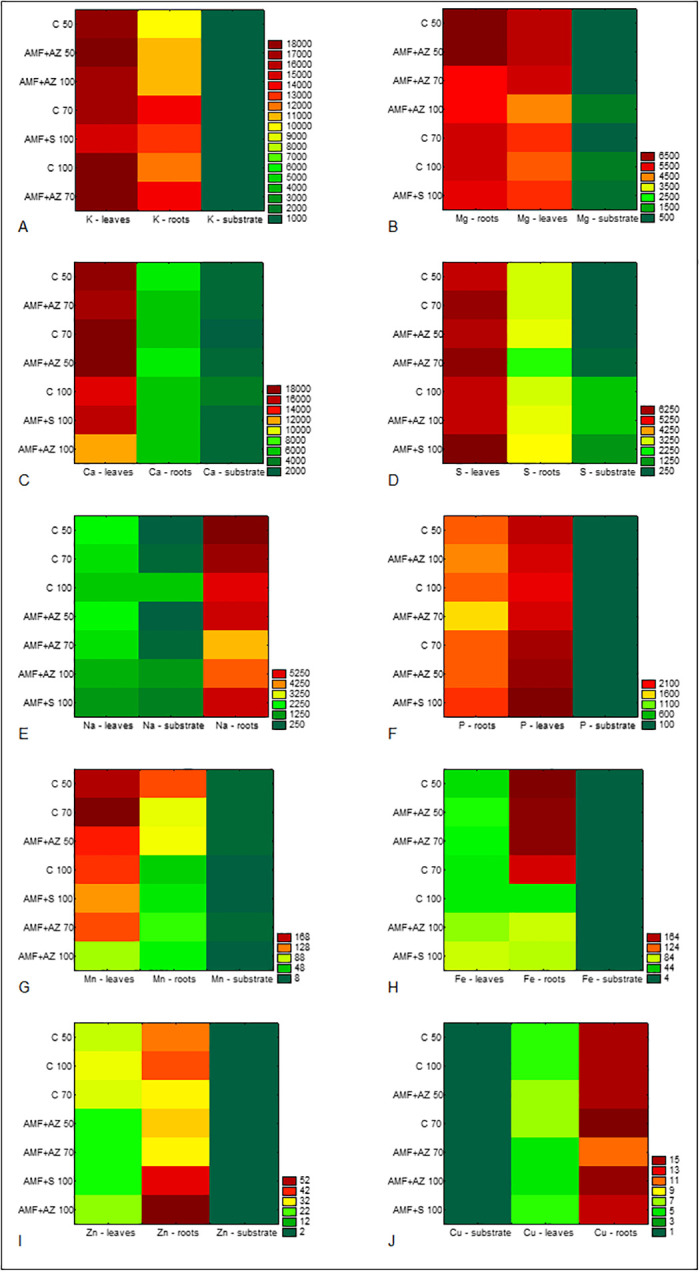
Heat map plots of elements content in soil/plant system under different substrate treatments: Potassium (A), magnesium (B), calcium (C), sulfur (D), sodium (E), phosphorus (F), manganese (G), iron (H), zinc (I), and copper (J). Green colors indicate that element contents were less than the means, while red colors indicate that element contents were higher than the means. C 50 –peat:sand ratio 50:50 (v:v) without inoculation; C 70 –peat:sand ratio 70:30 (v:v) without inoculation; C 100 –peat:sand ratio 100:0 (v:v) without inoculation; AMF + AZ 50 –peat:sand ratio 50:50 (v:v) inoculated with arbuscular mycorrhizal fungi (AMF) and *Azospirillum brasilense* (AZ), AMF + AZ 70 –peat:sand ratio 70:30 (v:v) inoculated with AMF and AZ, AMF + AZ 100 –peat:sand ratio 100:0 (v:v) inoculated with AMF and AZ; AMF + S– 100 peat:sand ratio 100:0 (v:v) inoculated with AMF and *Saccharothrix tamanrassetensis* (S).

The mineral content in substrates and tomato seedlings was significantly affected by substrate composition and microbial inoculation. The lowest potassium root:shoot ratio occurred in plants collected from C 50 and AMF + AZ 50 treatments and was equal to 0.56 and 0.55, respectively, whereas the highest was determined for plants in the C 70 substrate (0.82) (Fig 7). Potassium was accumulated in tomato leaves in significantly higher amounts than in the roots, which was visually confirmed with the use of heat maps (Fig 8A). Leaves of plants collected from AMF + AZ 50 and AMF + AZ 70 treatments contained the highest total K level, whereas roots of plants grown in the C 50 treatment had the lowest (Table 4). The correlation coefficients between elements in plants and soil are presented in Table 1.

The leaves sampled from plants grown in C 50, C 70, AMF + AZ 50, and AMF + AZ 70 treatments contained the highest amounts of Ca, whereas the lowest was determined in plant roots in AMF + AZ 100 and AMF + S 100 treatments (Table 4, Fig 8B). The Ca root:shoot ratio was 0.30 for the C 70 treatment (the lowest value) and 0.43 for the AMF + AZ 100 treatment (the highest value) (Fig 7).

The distribution of Mg was different among plant parts and treatments, and the root:shoot ratios were in the range from 0.69 (C 70) to 1.27 (AMF + AZ 100) (Fig 7). Roots of tomato seedlings from the control treatments contained higher Mg levels than leaves, whereas an inverse relationship was noted for the AMF + AZ 70 treatment. In the remaining treatments, differences between roots and leaves concerning Mg content were not significant. The highest Mg amount was in roots of plants grown in C 50 and AMF + AZ 50 substrates (Table 4, Fig 8C).

The sulfur root:shoot ratio was the highest in plants collected from the C 70 treatment (2.09) followed by the AMF + AZ 100 and AMF + AZ 50 (1.89 and 1.80, respectively) (Fig 7). Tomato leaves collected from all substrates contained a higher amount of sulfur than roots, especially those sampled from the AMF + AZ 70 and AMF + S 100 treatments (Table 4, Fig 8D).

The root:shoot accumulation ratios were 0.26, 0.28, and 0.40 for C 70, AMF + AZ 50, and AMF + AZ 100, respectively, whereas its value was close to 3 for the remaining treatments (Fig 7). Tomato seedlings accumulated Na in roots, especially those from the C 70 treatment (Table 4, Fig 8E).

P accumulated in leaves, especially in seedlings collected from the AMF + S 100 treatment, followed by the AMF + AZ 50 and C 70 treatments. The P content was lower in the roots than leaves. Moreover, differences between treatments were not statistically significant, with the root:shoot ratio below 1 (Table 5, Figs 7 and 8F).

**Table 5:** Effects of soil microorganisms application to substrates of different organic matter content on potassium, calcium, manganese, sulphur, and sodium content in leaves and roots of tomato transplants.

Treatment	Plant part	K	Ca	Mg	S	Na
		(mg kg^–1^ DW)				
C 50*	Leaves	17413 ± 462 fg**	17752 ± 94 ef	5825 f-g	5566 ± 95 c	2228 ± 63 d
	Roots	9725 ± 346 a	6806 ± 112 b	7180 h	3027 ± 274 b	6822 ± 40 h
C 70	Leaves	16206 ± 436 ef	18436 ± 215 f	4952 a-d	6331 ± 217 de	1787 ± 35 bc
	Roots	13337 ± 296 cd	5500 ± 393 ab	5712 e-g	3182 ± 393 b	6199 ± 119 g
C 100	Leaves	18245 ± 2120 fg	14532 ± 406 d	4537 ab	5735 ± 406 c	1737 ± 109 bc
	Roots	11935 ± 985 a-c	5359 ± 443 a	5655 c-g	3154 ± 443 b	5134 ± 424 f
AMF + AZ 50	Leaves	18646 ± 602 g	18720 ± 767 f	5626 e-h	5974 ± 769 c	2028 ± 35 cd
	Roots	10267 ± 859 ab	6358 ± 58 ab	7168 h	3251 ± 71 b	5297 ± 71 f
AMF + AZ 70	Leaves	18891 ± 292 g	16609 ± 1355 e	5958 g	6509 ± 1357 e	1834 ± 150 b-d
	Roots	13216 ± 987 cd	5563 ± 85 ab	5138 b-e	2440 ± 85 a	3955 ± 29 f
AMF + AZ 100	Leaves	14635 ± 234 de	11865 ± 97 c	4293 a	5656 ± 97 c	1489 ± 80 ab
	Roots	10242 ± 769 ab	5122 ± 324 a	5021 a-e	3293 ± 324 b	4318 ± 106 f
AMF + S 100	Leaves	16075 ± 234 ef	15050 ± 173 d	4872 a-c	7029 ± 173 f	1156 ± 90.0 a
	Roots	12378 ± 570 b-d	5025 ± 286 a	5470 c-g	3513 ± 286 b	5305 ± 23.0 f
Source of variation						
Treatment (T)		***	***	***	***	***
Plant part (P)		***	***	***	***	***
T×P		***	***	***	***	***

*C 50 –peat:sand ratio 50:50 (v:v) without inoculation; C 70 –peat:sand ratio 70:30 (v:v) without inoculation; C 100 –peat:sand ratio 100:0 (v:v) without inoculation; AMF + AZ 50 –peat:sand ratio 50:50 (v:v) inoculated with arbuscular mycorrhizal fungi (AMF) and Azospirillum brasilense (AZ), AMF + AZ 70 –peat:sand ratio 70:30 (v:v) inoculated with AMF and AZ, AMF + AZ 100 –peat:sand ratio 100:0 (v:v) inoculated with AMF and AZ; AMF + S– 100 peat:sand ratio 100:0 (v:v) inoculated with AMF and Saccharothrix tamanrassetensis (S).

**Means within a column, followed by different letters are significantly different, with comparisons performed using Tukey’s HSD test. Levels of significance:

*** P ≤ 0.001.

The leaf samples collected from the C 70 and C 50 treatments contained the highest P amount, as well as the root samples collected from the AMF + AZ 50 and C 70 treatments (Table 5, Fig 7). Similar to P, Mn content was higher in tomato leaves than roots, with the root:shoot accumulation ratio in the range from 0.37 (C 100) to 0.76 (C 50) (Fig 8F).

The substrates of control treatments, composed of peat:sand at the ratio 50:50 (v:v) and 70:50 (v:v), as well as the AMF + AZ 70 and AMF + AZ 100 treatments, contained significantly higher levels of soluble Fe compared to the remaining treatments (Table 3). Fe content was the highest in tomato seedling roots sampled in the treatments C 50, AMF + AZ 100, and AMF + AZ 70, followed by C 70. In general, roots and leaves of tomatoes collected from the remaining substrates contained significantly lower amounts of Fe, which were primarily not differentiated between treatments or plant parts (Table 6, Fig 7). The root:shoot Fe ratio was also distinguished among treatments and was in the range from 0.27 (C 70) to 3.86 (C 50) (Fig 8H).

**Table 6:** Effects of soil microorganisms application to substrates of different organic matter on phosphorus, manganese, iron, zinc, and copper content in leaves and roots of tomato transplants.

Treatment	Plant part	P	Mn	Fe	Zn	Cu
		(mg kg^–1^ DW)				
C 50*	Leaves	2382 ± 99 e–g**	174.29 ± 3.51 h	50.49 ± 0.31 a	24.57 ± 1.11 bc	5.65 ± 0.02 a
	Roots	1826 ± 101 a–c	132.42 ± 1.93 fg	194.86 ± 9.14 f	37.05 ± 1.29 f	14.66 ± 0.43 cd
C 70	Leaves	2592 ± 14 f–h	206.46 ± 3.89 i	53.32 ± 1.42 ab	27.17 ± 1.13 cd	6.29 ± 0.13 a
	Roots	1876 ± 213 a–d	99.21 ± 1.11 e	151.68 ± 19.8 e	30.61 ± 1.98 de	16.97 ± 0.20 e
C 100	Leaves	2146 ± 106 c–e	137.24 ± 6.83 g	54.38 ± 2.32 ab	29.97 ± 1.62 de	5.64 ± 1.13 a
	Roots	1851 ± 153 a–c	50.46 ± 4.17 a	52.62 ± 4.34 ab	38.33 ± 3.16 f	14.83 ± 1.22 cd
AMF + AZ 50	Leaves	2652 ± 95 gh	142.01 ± 5.48 g	62.05 ± 1.24 b–d	19.57 ± 1.30 a	6.28 ± 0.13 a
	Roots	1886 ± 7 a–d	102.84 ± 1.80 e	82.01 ± 2.12 d	33.8 ± 1.737 ef	14.79 ± 0.41 cd
AMF + AZ 70	Leaves	2225 ± 240 de	134.73 ± 0.74 g	58.68 ± 2.03 a–c	18.20 ± 0.60 a	4.76 ± 0.22 a
	Roots	1529 ± 12 a	69.31 ± 2.21 c	187.31 ± 7.64 f	31.15 ± 1.92 de	10.47 ± 0.74 b
AMF + AZ 100	Leaves	2279 ± 13 e–g	83.61 ± 3.24 d	72.38 ± 0.92 b–d	22.33 ± 1.74 ab	4.64 ± 0.14 a
	Roots	1782 ± 63 ab	60.55 ± 1.37 bc	187.84 ± 5.58 f	60.66 ± 0.90 h	15.19 ± 0.66 d
AMF + S 100	Leaves	2934 ± 17 h	123.25 ± 0.26 f	80.43 ± 0.26 d	19.69 ± 0.20 a	5.22 ± 0.17 a
	Roots	1917 ± 174 b–d	58.37 ± 2.02 ab	78.44 ± 4.65 cd	44.21 ± 1.30 g	13.42 ± 0.35 c
Source of variation						
Treatment (T)		***	***	***	***	***
Plant part (P)		***	***	***	***	***
T×P		***	***	***	***	***

*C 50 –peat:sand ratio 50:50 (v:v) without inoculation; C 70 –peat:sand ratio 70:30 (v:v) without inoculation; C 100 –peat:sand ratio 100:0 (v:v) without inoculation; AMF + AZ 50 –peat:sand ratio 50:50 (v:v) inoculated with arbuscular mycorrhizal fungi (AMF) and Azospirillum brasilense (AZ), AMF + AZ 70 –peat:sand ratio 70:30 (v:v) inoculated with AMF and AZ, AMF + AZ 100 –peat:sand ratio 100:0 (v:v) inoculated with AMF and AZ; AMF + S– 100 peat:sand ratio 100:0 (v:v) inoculated with AMF and Saccharothrix tamanrassetensis (S).

**Means within a column, followed by different letters are significantly different, with comparisons performed using Tukey’s HSD test. Levels of significance:

*** P ≤ 0.001.

Roots of plants grown in the AMF + AZ 100 substrate contained the highest level of Zn, followed by AMF + S 100, with a root:shoot ratio of 0.51 and 1.72, respectively. Analysis of the chemical composition of leaves shoved significantly higher Zn content in leaves of plants collected from the treatments C 50 –C 70 compared to those collected from the microbiota application treatments (Table 6, Figs 7 and 8I).

Leaves of tomatoes from all treatments did not differ in Cu content, which was generally lower than that found in roots. Different concentrations of Cu characterized roots of investigated treatments, with the highest values in plant roots from the C 70 treatment and the lowest from the AMF + AZ 100 treatment (Table 6, Fig 8J). The root:shoot accumulation ratio was 0.33, 0.42, and 0.43 for plants cultivated in the substrate AMF + AZ 50, and AMF + AZ 100, and C 70, respectively, whereas its value was close to 2.5 for the remaining treatments (Fig 7).

## Discussion

### Substrate characteristics

Substrate analysis after tomato cultivation in the present research confirmed the crucial significance of organic matter content for its physical and chemical characteristics. The highest total N, C-organic, sum of alkaline cations, and salinity, and the lowest pH was in substrates composed of peat, and the inoculation with AMF and bacteria had slight effect on these parameters. The substrates exhibited significantly different mineral composition after tomato cultivation. Even under conditions of high salinity, the mineral soluble forms were not reduced, especially in inoculated treatments. The uptake of N, P, Mg, Ca, Mn, and Fe were enhanced in inoculated tomato [53]. The chemical properties of the substrate influenced the amount of biomass produced [54,55].

The substrate composition affects the bioavailability of elements, both necessary and potentially toxic for plants. The capacity of the sorption complex plays a key role in the release of ions into the soil solution, associated with changes in the salinity level and soil pH. The capacity of the sorption complex in the tested substrates ranged from 364 to 1524 mM kg^-1^, and was proportional to the share of the organic component in the substrate. The lowest values were found in treatments C 50, C 70, AMF + AZ 50, and AMF + AZ 70, while the highest values in all treatments with peat only. Despite significant differences in this parameter from individual treatments, it was not reflected in the level of saturation of the sorption complex with alkaline cations, which ranged from 87.9% to 91.5%. Under conditions of maintaining the optimal fertilization and irrigation, the capacity of the sorption complex does not influence the development of plants [56]. A more important parameter is the saturation of the sorption complex with alkaline cations, which indicates the potential negative impact of hydrogen ions on plant growth. The pH reaction is related to the level of saturation of the sorption complex. Despite the large differences in the content of organic fraction in the substrates and the capacity of the sorption complex, slight differences in the substrate pH occurred after the experiment. No translation was found between the amount of organic materials in the substrate and its strategic properties. This could be caused by the short duration of the experiment. The content of elements was low and in the treatments with a small amount of organic component in the substrate, they could be assessed as deficient for plants [54]. This is especially true of P and K. The content of micronutrients and potentially toxic elements in no case indicated a threat to plants [57]. A negative correlation was found between the reaction and the content of most elements in the medium, and a positive correlation between the content of organic C and the amount of assimilable forms of the elements. The exceptions were Fe and Mn with opposite relationships.

### Plant weight

Shoot and root DW and leaf area of tomatoes grown in a low P soil-sand mix were higher in mycorrhizal than in nonmycorrhizal plants [58]. Ribaudo et al. [59] determined that tomato inoculation with *Azospirillum brasilense* FT326 significantly enhanced the root and shoot weight. Increase in biomass weight was correlated with increased absorption of mineral elements, such as N, P, K, Ca, and Mg which was noticed under inoculation with single and combined bacterial strains, i.e., *Azotobacter* or *Azospirillum*. Similar dependence was found in the present research.

### Root colonization

The mycorrhizosphere and sporosphere bacteria can boos germination and/or improving the growth of extraradical mycelium, the fine absorbing network of hyphae extend around the roots [60]. Colonization was confirmed in all treatments inoculated by AMF or *A*. *brasilense*. The colonization rate was increased in peat (100%), which showed a higher ability of AMF to develop symbiosis, contrary to the sand. Paranavithana et al. [61] found the higher C soil content corresponded to the higher AMF colonization in rice. The results showed the same correlation in the rate of colonization and C content. AMF colonizing plants could support rhizodeposition in soil, and as a result mycorrhiza increased plant C sequestration [62]. This effect improves soil amelioration of degraded soils by higher organic matter deposition and sorption of water and nutrients.

*Saccharothrix* spp. is producing dithiolopyrolone derivates with antifungal activity [21]. Such studies were background for our goal of confirming whether *Saccharothrix* could negatively affect formation of mycorrhizal symbiosis. Results have shown good development of AMF in co-inoculation with *S*. *tamanrassetensis* and other positive effects on some others analytical parameters.

### Physiological parameters

NDVI relates to the chlorophyll content in leaves and provides information on the photosynthetic activity [63,64]. The positive impact of microorganism was not documented in our study. However, this data was in line with Nogales et al. [65] results on *F*. *mosseae*-inoculated grapevine plants, which showed the decrease in NDVI after 3 months growth in Cu-contaminated soil. The Cu-contamination created stressful soil conditions, which could develop a similar chain of basic physiological reactions. The quantum yield of photosynthesis is a widely used measurement in many studies [66]. Quantum yield and NDVI values represent better nitrogen plant management. Many studies [67] have shown a positive correlation to the increased N fertilization in tomatoes. However, we did not confirm this effect in AMF or AZ treatments. Although in AMF it could be expected because most AMF are phosphorus plant uptake enhancers, *Azospirillum* is a typical N-fixing bacterium. The possibility of quantitative and time-dependent limitation of microbial colony formation [68] in mutual AMF and *Azospirillum* presence in the substrate could lead to this result.

### Analyses of stress biomarkers

Plants grown in substrate reflecting degraded soil conditions are under stress conditions modifying root system growth, functioning, and mineral absorption effectiveness. PGPM can partially compensate the chemical fertilizers, especially in tomatoes, a highly mycorrhizal-dependent crop [69]. The present study showed the new aspect of this relationship because tomato grown in substrate with AMF and *Azospirillum brasilense* showed the lowest antioxidant activity. In contrast, those inoculated with AMF and *S*. *tamanrassetensis* showed the highest antioxidant activity in both roots and leaves. The present study confirmed the organ-dependent polyphenol concentration. Inculet et al. [70] showed long-term effects of PGPM inoculation on tomato growth, yield, and fruit polyphenol content and antioxidant activity. In general, plants exhibit an increased synthesis of polyphenols under abiotic stress, including suboptimal soil conditions [71]. Abiotic stresses create osmotic stress [72], oxidative damage [73], and reactive oxygen species (ROS) [74], that lead to numerous physio-molecular changes, including a decrease in photosynthetic activities [75], DNA, protein and membrane damages, and nutritional imbalance in plants [76] and ultimately affect plant growth and productivity [77]. Nevertheless, to adjust stress, stress-induced plant evolved mechanisms to enhance the concentration of the majority of polyphenols [78,79] and detoxify the ROS. Phenolic compounds have high antioxidant activity [80] that can scavenge reactive oxygen species [81], and this observation lies behind the high level of total phenols in the C 50 treatment, reflecting the eroded soil without microorganisms amendment. Soil microbes transform phenolics into compounds, which help in element mineralization [9]. Phenolic compounds improve nutrient uptake through chelation of metallic ions, enhanced active absorption sites, and soil porosity with accelerated mobilization of many elements [82]. This observation can explain a positive correlation between antioxidant activity, total phenolics, and some elements mentioned in the present study which were corroborative to the previous findings [83].

Another aspect of the present research is the translocation of mineral elements to shoots. POX catalyzes lignin formation and establishes structural barriers by producing reactive oxygen and nitrogen species [84]. This explains the high activity of GPOX in roots of tomato seedlings, confirmed for all treatments, except for C 50, where stress conditions could interrupt this line of defense. Species of *Rhizobacteria*, *Azospirillum*, and *Pseudomonas* play a significant role in tomato competition for nutrients or space [85]. Thus, inoculation of tomato with *A*. *brasilense* could cause increased GPOX activity in inoculated treatments with the substrate with a low amount of organic matter (AMF + AZ 50 and AMF + AZ 70). Accordingly, Islam et al. [86] stated that PGPB could activate plant antioxidant defense by regulating the activity of superoxide dismutase, catalase, and peroxidase, the key enzymes that deactivate over produced reactive oxygen species.

### Element concentration in plant tissues

We analyzed the substrate content of available forms of nutrients and the uptake by tomato roots followed by translocation to shoots. Plants need to develop efficient strategies to enhance K uptake from the soil, which includes association with soil microbiota [87].

According to Meena et al. [88] the most effective K, P, and Zn-solubilizing bacteria belong to the genera *Azospirillum*, *Azotobacter*, and *Bacillus*. The results concerning potassium substrate/root/shoot translocation showed an effective K collection from the peat:sand substrate, accelerated by consortia AMF + AZ 50 and AMF + AZ 70. According to Singh et al. [89], K acquisition from soils with low soluble K concentration can be enhanced by mycorrhizal symbiosis. In the present research, the negative correlation observed for K in the substrate and in tomato leaves indicated the existence of microbial-assisted release and K uptake.

Positive effects of K on dry biomass are observed; however, increasing K application can also decrease the economic benefit because excess K can reduce the mobility of calcium [90]. The negative antagonism was confirmed for K and Ca in the aboveground tomato tissues in the presented research. Free Ca level in a plant tissue is a stress signaling factor, and Ca^2+^ plays a vital role in many functions. AMF selectively uptake K^+^ and Ca^2+^, which act as osmotic equivalents as they avoid the uptake of toxic Na^+^, especially in the saline soils [91]. However, in the present research, inoculation did not have a significant effect on Ca^2+^ absorption, which was dependent mainly on substrate composition. Root tissues accumulate a higher level of Na^+^ than shoots; moreover, in mycorrhizal roots Na^+^ may be compartmentalized in cell vacuoles and in AMF hyphae to prevent translocation to the shoots [92]. Indeed, tomato seedlings accumulated Na in roots in the conditions of the present experiment.

PGPM inoculation improved P, Mg, and Ca contents in plants. Several studies have shown that AMF helps in the P nutrition of plants to the extent of saving NPK fertilizer application with no adverse effect on growth and yield of tomatoes [93]. AMF and phosphate solubilizing bacteria (PSB) could interact synergistically because PSB solubilizes sparingly available P into orthophosphate such that AMF can absorb and transport it to the host plant [94]. Bacteria help to mineralize organic P in the soil by the synthesis of enzymes (phytases, phosphonoacetate hydrolases) [95]. Although there is some evidence concerning the effect of PGPM on P and K plant nutrition, the knowledge regarding the other elements is limited. PGPM also improved Fe concentration in tomato across all conditions, concerning low and high P availability and Mg under low P availability to plants [96]. Mg is involved in a wide range of physiological activities, including pigment synthesis, energy metabolism, and photosynthetic carbon fixation [97]. An interesting result was the higher Mg accumulation in roots and leaves of tomato in substrate composed of peat:sand 50:50 and 50:70 (v:v) inoculated with AMF + AZ, as compared to the other inoculants applied. AMF + S inoculation significantly increased Fe and S accumulation in tomato leaves. According to available references, total accumulation of Zn, Cu, and Fe was higher, but Na was lower in mycorrhizal tomatoes grown in low-P soil (Al-Karaki, 2000). Azcón et al. [98] determined in lettuce, that with high substrate availability of P, the other nutrients level decreased. However, with a low level of P, macro- and micronutrients increased. This observation can explain negative correlations between substrates P and Ca, Mg, and Mn in roots and leaves, and Fe in roots of tomato in the present research.

## Conclusion

The improved growth and nutrient acquisition in tomatoes demonstrated the potential of AMF and bacteria colonization for protecting plants cultivated in degraded soil conditions. The establishment of the symbiosis, observed with confocal microscopy, modified the substrate conditions and involved a continuous cellular and molecular dialogue between AMF, AMF + bacteria, and plants, which included the activation of different metabolic pathways. The most spectacular effects of microorganisms in supporting the plant fitness in the conditions reflecting degrading soil covered decreased antioxidant activity and phenolic compound level as compared to the non-mycorrhizal control. This finding corresponded to the higher tolerance to stressful conditions and enhanced uptake of some nutrients followed by DW increase. However, the consortia of plant growth promoting microorganisms acted the most effectively in substrate rich in organic matter, positively shaping the parameters characterizing tomato seedling fitness. The application pro-ecological methods to improve growth and nutritional quality of tomatoes definitely include the beneficial microorganisms dedicated to positively affect plant performance.

## Supporting information

S1 Fig Mycelial structures (m) of fungi are mutually surrounding root hairs (rh) of tomato showing development of „interface”for the mutual flow of water and nutrients through the fungal and root grid. The soil with higher organic matter has showed better netting. Figure was taken in sample at AMF + AZ 100 treatment. **1_2. Development of AMF spores (s) in root hairs (rh) area was also detected after inoculation in the treatment with low organic matter content (AMF + AZ 50)**. This confirms ability of AMF to colonize plant roots and form the propagative structures for further substrate colonizing. **1_3 A, 1_3B. Set up of symbiosis between AMF and tomato plants was described on arbuscules (a) structures found in root tissues**. The treatments with peat showed higher levels of arbuscules abundance. Both figures show the symbiosis created in sand:peat substrate 50:50, also (AMF + AZ 50). Bar = 20 μm.
(TIF)

S2 Fig Bacterial colonies (b) on tomato root hairs (rh) were found in all treatments with inoculation by *Azospirillum brasilense*. In sterile conditions of substrate were found only these bacterial colonies as abundant (treatment AMF + AZ 70). Bar = 20 μm.
(TIF)

S3 Fig Bacterial colonies (b) on tomato root hairs (rh) were found in all treatments with inoculation by *Azospirillum brasilense*. In sterile conditions of substrate were found only these bacterial colonies as abundant (treatment: AMF + AZ 100). Bar = 20 μm.
(TIF)

## References

[1] EU Environment. (2021). Soil and Land. https://ec.europa.eu/environment/soil/index_en.htm [Accessed February 17, 2021].

[2] Vimal SR, Singh JS, Arora NK, Singh S. Soil-plant-microbe interactions in stressed agriculture management: a review. Pedosphere. 2017;27(2):177–192. doi: 10.1016/S1002-0160(17)60309-6

[3] Onet A, Dincă LC, Grenni P, Laslo V, Teusdea AC, Vasile DL et al. Biological indicators for evaluating soil quality improvement in a soil degraded by erosion processes. J Soils Sedim. 2019;19(5):2393–2404. doi: 10.1007/s11368-018-02236-9

[4] Lehman MR, Cambardella CA, Stott DE, Acosta-Martinez V, Manter DK, Buyer JS et al. Understanding and Enhancing Soil Biological Health: The Solution for Reversing Soil Degradation. Sustainability. 2015;7:988–1027. doi: 10.3390/su7010988

[5] Saia S, Rappa V, Ruisi P, Abenavoli MR, Sunseri F, Giambalvo D et al. Soil inoculation with symbiotic microorganisms promotes plant growth and nutrient transporter genes expression in durum wheat. Front Plant Sci. 2015;6:815. doi: 10.3389/fpls.2015.00815 26483827 PMC4591488

[6] Saia S, Ruisi P, Fileccia V, Di Miceli G, Amato G, Martinelli F. Metabolomics suggests that soil inoculation with arbuscular mycorrhizal fungi decreased free amino acid content in roots of durum wheat grown under N-limited, P-rich field conditions. PLoS One. 2015;10(6):e0129591. doi: 10.1371/journal.pone.0129591 26067663 PMC4466249

[7] Szczałba M, Kopta T, Gastoł M, Sękara A. Comprehensive insight into arbuscular mycorrhizal fungi, *Trichoderma* spp. and plant multilevel interactions with emphasis on biostimulation of horticultural crops. J Appl Microbiol. 2019;127(3). doi: 10.1111/jam.14247 30844108

[8] Saia S, Tamayo E, Schillaci C, De Vita P. Arbuscular mycorrhizal fungi and nutrient cycling in cropping systems. In: Datta R, Meena RS, Pathan SI, Ceccherini MT, editors. Carbon and nitrogen cycling in soil. Singapore: Springer; 2020. p. 87–115.

[9] Raaijmakers JM, Paulitz TC, Steinberg C, Alabouvette C, Moënne-Loccoz Y. The rhizosphere: a playground and battlefield for soilborne pathogens and beneficial microorganisms. Plant Soil. 2009;321(1–2):341–361. doi: 10.1007/s11104-008-9568-6

[10] Syed S, Tollamadugu NP. Role of Plant Growth-Promoting Microorganisms as a Tool for Environmental Sustainability. In: Viswanath B, editor. Recent Developments in Applied Microbiology and Biochemistry. New York: Academic Press; 2019. p. 209–222.

[11] Hao L, Zhang Z, Hao B, Diao F, Zhang J, Bao Z et al. Arbuscular mycorrhizal fungi alter microbiome structure of rhizosphere soil to enhance maize tolerance to La. Ecotoxicol Environ Saf. 2021; 212:111996. doi: 10.1016/j.ecoenv.2021.111996 33545409

[12] Kleinert A, Benedito V, Morcillo R, Dames J, Cornejo-Rivas P, Zuniga-Feest A et al. Morphological and Symbiotic Root Modifications for Mineral Acquisition from Nutrient-Poor Soils. Soil Biology. 2018;85–142.

[13] Ditengou FA, Müller A, Rosenkranz M, Felten J, Lasok H, van Doorn MM et al. Volatile signalling by sesquiterpenes from ectomycorrhizal fungi reprogammes root architecture. Nat Commun. 2015;6:6279. doi: 10.1038/ncomms7279 25703994 PMC4346619

[14] Smith S.E., Smith F.A. Fresh perspectives on the roles of arbuscular mycorrhizal fungi in plant nutrition and growth. Mycologia. 2012;104:1–13. doi: 10.3852/11-229 21933929

[15] Seguel A, Cornejo P, Ramos A, Von Baer E, Cumming J, Borie F. Phosphorus acquisition by three wheat cultivars contrasting in aluminum tolerance growing in an aluminum-rich volcanic soil. Crop Pasture Sci. 2017;68:305–316. doi: 10.1071/CP16224

[16] Krishnamoorthy R, Kim K, Subramanian P, Senthilkumar M, Anandham R, Sa T. Arbuscular mycorrhizal fungi and associated bacteria isolated from salt-affected soil enhances the tolerance of maize to salinity in coastal reclamation soil. Agric Ecosyst Environ 2016; 231:233–239. doi: 10.1016/j.agee.2016.05.037

[17] Ruiz-Lozano JM, Porcel R, Calvo-Polanco M, Aroca R. Improvement of Salt Tolerance in Rice Plants by Arbuscular Mycorrhizal Symbiosis. In: Giri B et al., editors. Root Biology, Soil Biology. Springer International Publishing: 2018, p. 259–280.

[18] Jung SC, Martinez-Medina A, Lopez-Raez JA, Pozo MJ. Mycorrhiza-induced resistance and priming of plant defenses. J Chem Ecol. 2012;38:651–664. doi: 10.1007/s10886-012-0134-6 22623151

[19] Miozzi L, Vaira AM, Catoni M, Fiorilli V, Accotto GP, Lanfranco L. Arbuscular mycorrhizal symbiosis: plant friend or foe in the fight against viruses? Front Microbiol. 2019;10:1238. doi: 10.3389/fmicb.2019.01238 31231333 PMC6558290

[20] Kim B, Brown R, Labeda D, Goodfellow M. Reclassification of ‘*Dactylosporangium variesporum*’ as *Saccharothrix variisporea* corrig. (ex Tomita et al. 1977) sp. nov., nom. rev. Int J Syst Evol Microbiol. 2011;61(2):310–314. 20305067 10.1099/ijs.0.021857-0

[21] Merrouche R, Yekkour A, Lamari L, Zitouni A, Mathieu F, Sabaou N. Efficiency of *Saccharothrix algeriensis* NRRL B-24137 and its produced antifungal dithiolopyrrolones compounds to suppress *Fusarium oxysporum*-induced wilt disease occurring in some cultivated crops. Arabian J Sci Engin. 2017;42(6):2321–2327. doi: 10.1007/s13369-017-2504-4

[22] Pii Y, Mimmo T, Tomasi N, Terzano R, Cesco S, Crecchio C. Microbial interactions in the rhizosphere: beneficial influences of plant growth-promoting rhizobacteria on nutrient acquisition process. A review. Biol Fertil Soils. 2015;51(4):403–415. doi: 10.1007/s00374-015-0996-1

[23] Fukami J, Cerezin P, Hungria M. *Azospirillum*: benefits that go far beyond biological nitrogen fixation. AMB Express. 2018;8:1–12. doi: 10.1186/s13568-017-0531-x 29728787 PMC5935603

[24] Fukami J, Nogueira M, Araujo R, Hungria M. Accessing inoculation methods of maize and wheat with *Azospirillum brasilense*. AMB Express. 2016;6(3). doi: 10.1186/s13568-015-0171-y 26759120 PMC4710622

[25] Wallenstein MD. Managing and manipulating the rhizosphere microbiome for plant health: a systems approach. Rhizosphere. 2017;3:230–232. doi: 10.1016/j.rhisph.2017.04.004

[26] Kong W, Meldgin DR, Collins JJ, Lu T. Designing microbial consortia with defined social interactions. Nat Chem Biol. 2018;14:821–829. doi: 10.1038/s41589-018-0091-7 29942078

[27] Woo SL, Pepe O. Microbial consortia: promising probiotics as plant biostimulants for sustainable agriculture. Front Plant Sci. 2018;9:1801. doi: 10.3389/fpls.2018.01801 30564264 PMC6288764

[28] Barea JM. Future challenges and perspectives for applying microbial biotechnology in sustainable agriculture based on a better understanding of plant-microbiome interactions. J Soil Sci Plant Nutr. 2015;15:261–282. doi: 10.4067/S0718-95162015005000021

[29] Bidondo LF, Colombo R, Bompadre J, Benavides M, Scorza V, Silvani V et al. Cultivable bacteria associated with infective propagules of arbuscular mycorrhizal fungi. Implications for mycorrhizal activity. Appl Soil Ecol. 2016;105:86–90. doi: 10.1016/j.apsoil.2016.04.013

[30] Bitterlich M, Rouphael Y, Graefe J, Franken P. Arbuscular mycorrhizas: a promising component of plant production systems provided favorable conditions for their growth. Front Plant Sci. 2018;9:1329. doi: 10.3389/fpls.2018.01329 eCollection 2018. 30250477 PMC6139337

[31] Pedersen JF, Vogel KP, Funnell DL. Impact of reduced lignin on plant fitness. Crop Sci. 2005;45(3):812–819. doi: 10.2135/cropsci2004.0155

[32] Kiers ET, Denison RF. Inclusive fitness in agriculture. Philosophical Transactions of the Royal Society B: Biological Sciences. 2014;369(1642):20130367. doi: 10.1098/rstb.2013.0367 24686938 PMC3982668

[33] Hart M, Ehret DL, Krumbein A, Leung C, Murch S, Turi C et al. Inoculation with arbuscular mycorrhizal fungi improves the nutritional value of tomatoes. Mycorrhiza. 2015;25(5):359–376. doi: 10.1007/s00572-014-0617-0 25391485

[34] Ahmed B, Zaidi A, Khan MS, Rizvi A, Saif S, Shahid M. Perspectives of plant growth promoting rhizobacteria in growth enhancement and sustainable production of tomato. In: Zaidi A, Khan M., editors. Microbial Strategies for Vegetable Production. Cham. Springer. 2017. p. 125–149.

[35] Alabid I, Glaeser SP, Kogel KH. Endofungal bacteria increase fitness of their host fungi and impact their association with crop plants. Curr Issues Mol Biol. 2018;30(1):59–74. doi: 10.21775/cimb.030.059 30070651

[36] Martin-Rivilla H, Garcia-Villaraco A, Ramos-Solano B, Gutierrez-Manero FJ, Lucas J. A. Improving flavonoid metabolism in blackberry leaves and plant fitness by using the bioeffector *Pseudomonas fluorescens* N 21.4 and its metabolic elicitors: A biotechnological approach for a more sustainable crop. J Agric Food Chem. 2020;68(22):6170–6180. doi: 10.1021/acs.jafc.0c01169 32383861

[37] Inui Kishi RN, Galdiano Júnior RF, Val-Moraes SP, Kishi LT. Soil Microbiome and Their Effects on Nutrient Management for Plants. In: Kumar V, Kumar M, Sharma S, Prasad R, editors. Probiotics in Agroecosystem. Singapore: Springer. 2017. 10.1007/978-981-10-4059-7_6.

[38] Smith FA, Smith SE. How useful is the mutualism-parasitism continuum of arbuscular mycorrhizal functioning? Plant Soil. 2013;363:7–18. doi: 10.1007/s11104-012-1583-y

[39] O’Callaghan M. Microbial inoculation of seed for improved crop performance: issues and opportunities. Appl Microbiol Biotechnol. 2016;100(13):5729–5746. doi: 10.1007/s00253-016-7590-9 27188775 PMC4909795

[40] Shirling ET, Gottlieb D. Methods for characterization of *Streptomyces* species. Int J Sys Bacteriol. 1966;16(3):313–340. doi: 10.1099/00207713-16-3-313

[41] Nowosielski O. Methods of fertilization need estimation. Warsaw: PWRiL; 1974. p. 1–91.

[42] Schmidt SK, Sobieniak-Wiseman LC, Kageyama SA, Halloy SRP, Schadt CW. Mycorrhizal and Dark-Septate Fungi in Plant Roots Above 4270 Meters Elevation in the Andes and Rocky Mountains. Arct Antarc Alp. 2008;40(3):576–583, doi: 10.1657/1523-0430(07-068)[SCHMIDT]2.0.CO;2

[43] Diagne N, Escoute J, Lartaud M, Verdeil J, Franche C, Kane A et al. Uvitex2B: a rapid and efficient stain for detection of arbuscular mycorrhizal fungi within plant roots. Mycorrhiza. 2011;21(4):315–321. doi: 10.1007/s00572-010-0357-8 21225294

[44] Vierheilig H, Schweiger P, Brundrett M. An overview of methods for the detection and observation of arbuscular mycorrhizal fungi in roots. Physiol Plant. 2005;125:393–404. doi: 10.1111/j.1399-3054.2005.00564.x

[45] Giovannetti M, Mosse B. An evaluation of techniques for measuring vesicular arbuscular mycorrhizal infection in roots. New Phytol. 1980;84:489–500. doi: 10.1111/j.1469-8137.1980.tb04556.x

[46] Sarker U, Oba S. Color attributes, betacyanin, and carotenoid profiles, bioactive components, and radical quenching capacity in selected *Amaranthus gangeticus* leafy vegetables. Sci Rep. 2021;11(1):1–14. 34079029 10.1038/s41598-021-91157-8 PMC8172918

[47] Molyneux P. The use of the stable free radical diphenylpicrylhydrazyl (DPPH) for estimating antioxidant activity. Songklanakarin J Sci Technol. 2004;26:211–219.

[48] Djeridane A, Yousfi M, Nadjemi B, Boutassouna D, Stocker P, Vidal N. Antioxidant activity of some Algerian medicinal plants extracts containing phenolic compound. Food Chem. 2006;97:654–660. doi: 10.1016/j.foodchem.2005.04.028

[49] Lück H. Peroxidase. In: Bergmeyer HU, editor. Methoden der enzymatischen analyse. Germany: Weinheim; 1962. p. 895–897.

[50] Pasławski P, Migaszewski ZM. The quality of element determinations in plant materials by instrumental methods. Pol J Environ Stud. 2006;15(2a):154–164.

[51] Shapiro SS, Wilk MB. An analysis of variance test for normality (complete samples). Biometrika. 1965;52:591–611. doi: 10.2307/2333709

[52] Levene H. Robust tests for equality of variances. In: Contributions to probability and statistics Essays in honor of Harold Hotelling. 1961.p. 279–292.

[53] Balliu A, Sallaku G, Rewald B. AMF inoculation enhances growth and improves the nutrient uptake rates of transplanted, salt-stressed tomato seedlings. Sustainability. 2015;7(12):15967–15981. doi: 10.3390/su71215799

[54] Niemiec M, Chowaniak M, Zuzek D, Komorowska M, Mamurovich GS, Kodirov Gafurovich K et al. Evaluation of the chemical composition of soil as well as vine leaves and berries from the selected commercial farms in the republic of Tajikistan. J Elementol. 2020;25(2):675–686. doi: 10.5601/jelem.2019.24.4.1810

[55] Rashidov N, Chowaniak M, Niemiec M. Assessment of the impact of differences in fertilization on selected yield indices for grapes in the Sughd Region of Tajikistan. J Elementol. 2020;25(4):1257–1268. doi: 10.5601/jelem.2019.24.2.1863

[56] Kremenetskaya I, Ivanova L, Chislov M, Zvereva I, Vasilieva T, Marchevskaya V et al. Physicochemical transformation of expanded vermiculite after long-term use in hydroponics. Appl Clay Sci. 2020;198(15):105839. doi: 10.1016/j.clay.2020.105839

[57] Niemiec M, Chowaniak M, Sikora J, Szeląg-Sikora A, Grodek-Szostak Z, Komorowska M. Selected properties of soils for long-term use in organic farming. Sustainability. 2020;12(6):2509. doi: 10.3390/su12062509

[58] Al-Karaki GN. Growth of mycorrhizal tomato and mineral acquisition under salt stress. Mycorrhiza. 2000;10(2):51–54. doi: 10.1007/s005720000055

[59] Ribaudo CM, Krumpholz EM, Cassán FD, Bottini R, Cantore ML, Cura JA. *Azospirillum* sp. promotes root hair development in tomato plants through a mechanism that involves ethylene. J Plant Growth Regul. 2006;25:175–185. doi: 10.1007/s00344-005-0128-5

[60] Battini F, Cristani C, Giovannetti M, Agnolucci M. Multifunctionality and diversity of culturable bacterial communities strictly associated with spores of the plant beneficial symbiont Rhizophagus intraradices. Microbiol Res. 2016;183:68–79. doi: 10.1016/j.micres.2015.11.012 26805620

[61] Paranavithana TM, Marasinghe S, Perera GAD, Ratnayake RR. Effects of crop rotation on enhanced occurrence of arbuscular mycorrhizal fungi and soil carbon stocks of lowland paddy fields in seasonaly dry tropics. Paddy Water Environ. 2020;19(1):217–226. doi: 10.1007/s10333-020-00833-4

[62] Zho J, Zang HD, Loeppmann S, Gube M, Kuzyakov Y, Pausch J. Arbuscular mycorrhiza enhances rhizodeposition and reduces the rhizosphere priming effect on the decomposition of soil organic matter. Soil Biol Biochem. 2020;140:107641. doi: 10.1016/j.soilbio.2019.107641

[63] Oliveira T, Carvalho L, Oliveira L, Lacerda W, Acerbi FJr. NDVI time series for mapping phonological variability of forests across the cerrado biome in Minas Gerais, Brazil. In: Ed. Zhang X, editor. *Phenology and Climate Change*. Shanghai, China: In Tech; 2012. p. 253–272.

[64] Cristiano P, Madanes N, Campanello P, di Francescantonio D, Rodríguez S, Zhang Y et al. High NDVI and potential canopy photosynthesis of South American subtropical forests despite seasonal changes in leaf area index and air temperature. Forests. 2014; 5(2), 287–308. doi: 10.3390/f5020287

[65] Nogales A, Santos E, Abreu M, Arán D, Victorino G, Pereira H et al. Mycorrhizal inoculation differentially affects grapevine’s performance in copper contaminated and non-contaminated soils. Front. Plant Sci. 2019;9. doi: 10.3389/fpls.2018.01906 30740120 PMC6355709

[66] Hogewoning S, Wientjes E, Douwstra P, Trouwborst G, van Ieperen W, Croce R et al. Photosynthetic quantum yield dynamics: from photosystems to leaves. Plant Cell. 2012;24(5):1921–1935. doi: 10.1105/tpc.112.097972 22623496 PMC3442578

[67] Ihuoma SO, Madramootoo CA. Narrow-band reflectance indices for mapping the combined effects of water and nitrogen stress in field grown tomato crops. Biosys Eng. 2020;192:133–143. doi: 10.1016/j.biosystemseng.2020.01.017

[68] Vazquez MM, Cesar S, Azcon R, Barea JM. Interactions between arbuscular mycorrhizal fungi and other microbial inoculants (*Azospirillum*, *Pseudomonas*, *Trichoderma*) and their effects on microbial population and enzyme activities in the rhizosphere of maize plants. Appl Soil Ecol. 2000;15(3):261–272. doi: 10.1016/S0929-1393(00)00075-5

[69] Sellitto VM, Golubkina NA, Pietrantonio L, Cozzolino E, Cuciniello A, Cenvinzo V et al. Tomato yield, quality, mineral composition and antioxidants as affected by beneficial microorganisms under soil salinity induced by balanced nutrient solutions. Agriculture. 2019;9(5):110. doi: 10.3390/agriculture9050110

[70] Inculet C-S, Mihalache G, Sellitto VM, Hlihor R-M, Stoleru V. The effects of a microorganisms-based commercial product on the morphological, biochemical and yield of tomato plants under two different water regimes. Microorganisms. 2019;7(12):706. doi: 10.3390/microorganisms7120706 31888271 PMC6955974

[71] Sharma A, Shahzad B, Rehman A, Bhardwaj R, Landi M, Zheng B. Response of phenylpropanoid pathway and the role of polyphenols in plants under abiotic stress. Molecules. 2019;24(13):2452. doi: 10.3390/molecules24132452 31277395 PMC6651195

[72] Sarker U, Oba S. The response of salinity stress-induced A. tricolor to growth, anatomy, physiology, non-enzymatic and enzymatic antioxidants. Front Plant Sci. 2020;11. doi: 10.3389/fpls.2020.559876 33178233 PMC7596248

[73] Achten WM, Maes W, Reubens B, Mathijs E, Singh VP, Verchot L et al. Biomass production and allocation in *Jatropha curcas* L. seedlings under different levels of drought stress. Biomass Bioenergy. 2010;34(5):667–676.

[74] Sarker U, Oba S. Catalase, superoxide dismutase and ascorbate-glutathione cycle enzymes confer drought tolerance of *Amaranthus tricolor*. Sci rep. 2018;8(1):1–12. doi: 10.1038/s41598-017-17765-5 30405159 PMC6220278

[75] Sarker U, Oba S. Response of nutrients, minerals, antioxidant leaf pigments, vitamins, polyphenol, flavonoid and antioxidant activity in selected vegetable amaranth under four soil water content. Food chem. 2018;252:72–83. doi: 10.1016/j.foodchem.2018.01.097 29478565

[76] Sarker U, Islam MT, Oba S. Salinity stress accelerates nutrients, dietary fiber, minerals, phytochemicals and antioxidant activity in *Amaranthus tricolor* leaves. PLos One. 2018;13(11):e0206388. doi: 10.1371/journal.pone.0206388 30383779 PMC6211690

[77] Sarker U, Oba S. Salinity stress enhances color parameters, bioactive leaf pigments, vitamins, polyphenols, flavonoids and antioxidant activity in selected *Amaranthus* leafy vegetables. J Sci Food Agric. 2019;99(5):2275–2284. doi: 10.1002/jsfa.9423 30324618

[78] Sarker U, Oba S. Augmentation of leaf color parameters, pigments, vitamins, phenolic acids, flavonoids and antioxidant activity in selected *Amaranthus tricolor* under salinity stress. Scientific reports. 2018;8(1): 1–9. doi: 10.1038/s41598-017-17765-5 30120319 PMC6098045

[79] Sarker U, Oba S. Drought stress enhances nutritional and bioactive compounds, phenolic acids and antioxidant capacity of *Amaranthus* leafy vegetable. BMC Plant biology. 2018;18(1):1–15. doi: 10.1186/s12870-017-1213-1 30367616 PMC6203965

[80] Sarker U, Hossain MN, Iqbal MA, Oba S. Bioactive components and radical scavenging activity in selected advance lines of salt-tolerant vegetable amaranth. Frontiers in Nutrition. 2020;7. doi: 10.3389/fnut.2020.587257 33330589 PMC7734134

[81] Sarker U, Oba S. Polyphenol and flavonoid profiles and radical scavenging activity in leafy vegetable *Amaranthus gangeticus*. BMC Plant Biology. 2020;20(1):1–12. doi: 10.1186/s12870-019-2170-7 33138787 PMC7607633

[82] Seneviratne G, Jayasinghearachchi HS. Mycelial colonization by bradyrhizobia and azorhizobia. J Biosci. 2003;28:243–247. doi: 10.1007/BF02706224 12711817

[83] Sarker U, Oba S. Nutritional and bioactive constituents and scavenging capacity of radicals in *Amaranthus hypochondriacus*. Sci Rep. 2020;10(1):1–10. doi: 10.1038/s41598-019-56847-4 33203902 PMC7673121

[84] Marjamaa K, Kukkola EM, Fagerstedt KV. The role of xylem class III peroxidases in lignification. J Exp Bot. 2009;60:367–376. doi: 10.1093/jxb/ern278 19264758

[85] Spadaro D, Droby S. Development of biocontrol products for postharvest diseases of fruit: the importance of elucidating the mechanisms of action of yeast antagonists. Trends Food Sci Technol. 2016;47:39–49. doi: 10.1016/j.tifs.2015.11.003

[86] Islam F, Yasmeen T, Arif MS, Ali S, Ali B, Hameed S et al. Plant growth promoting bacteria confer salt tolerance in *Vigna radiata* by up-regulating antioxidant defense and biological soil fertility. Plant Growth Regul. 2015;80:23–36. doi: 10.1007/s10725-015-0142-y

[87] Nanjundappa A, Bagyaraj DJ, Saxena AK, Kumar M, Chakdar H. Interaction between arbuscular mycorrhizal fungi and *Bacillus* spp. in soil enhancing growth of crop plants. Fungal Biol Biotechnol. 2019;6:23. doi: 10.1186/s40694-019-0086-5 31798924 PMC6882151

[88] Meena VS, Bahadur I, Maurya BR, Kumar A, Meena RK, Meena SK et al. Potassium-solubilizing microorganism in evergreen agriculture: an overview. In: Meena V, Maurya B, Verma J, Meena R, editors. Potassium Solubilizing Microorganisms for Sustainable Agriculture. New Delhi:Springer; 2016.

[89] Singh NP, Singh RK, Meena VS, Meena RK. Can we use maize (*Zea mays*) rhizobacteria as plant growth promoter. Vegetos. 2015;28(1):86–99. doi: 10.5958/2229-4473.2015.00012.9

[90] Liu J, Hu T, Feng P, Wang L, Yang S. Tomato yield and water use efficiency change with various soil moisture and potassium levels during different growth stages. PLoS One. 2019;14(3):e0213643. doi: 10.1371/journal.pone.0213643 30917147 PMC6436690

[91] Hammer EC, Nasr H, Pallon J, Olsson PA, Wallander H. Elemental composition of arbuscular mycorrhizal fungi at high salinity. Mycorrhiza. 2011;21:117–129. doi: 10.1007/s00572-010-0316-4 20499112

[92] Estrada B, Aroca R, Maathuis FJM, Barea JM, Ruiz-Lozano JM. Arbuscular mycorrhizal fungi native from a Mediterranean saline area enhance maize tolerance to salinity through improved ion homeostasis. Plant Cell Environ. 2013;36:1771–1782. doi: 10.1111/pce.12082 23421735

[93] Ziane H, Meddad-Hamza A, Beddiar A, Gianinazzi S. Effects of arbuscular mycorrhizal fungi and fertilization levels on industrial tomato growth and production. Int J Agric Biol. 2017;19(2):341–347. doi: 10.17957/IJAB/15.0287

[94] Nacoon S, Jogloy S, Riddech N, Mongkolthanaruk W, Kuyper TW, Boonlue S. Interaction between phosphate solubilizing bacteria and arbuscular mycorrhizal fungi on growth promotion and tuber inulin content of *Helianthus tuberosus* L. Sci Rep. 2020;10:1–10. doi: 10.1038/s41598-019-56847-4 32188930 PMC7080738

[95] Sharma S, Compant S, Ballhausen MB, Ruppel S, Franken P. The interaction between Rhizoglomus irregulare and hyphae attached phosphate solubilizing bacteria increases plant biomass of *Solanum lycopersicum*. Microbiol Res. 2020;240:126556. doi: 10.1016/j.micres.2020.126556 32683279

[96] Saia S, Aissa E, Luziatelli F, Ruzzi M, Colla G, Ficca AG et al. Growth-promoting bacteria and arbuscular mycorrhizal fungi differentially benefit tomato and corn depending upon the supplied form of phosphorus. Mycorrhiza. 2020;30(1):133–147. doi: 10.1007/s00572-019-00927-w 31823026

[97] Gransee A, Führs H. Magnesium mobility in soils as a challenge for soil and plant analysis, magnesium fertilization and root uptake under adverse growth conditions. Plant Soil. 2013;368:5–21. doi: 10.1007/s11104-012-1567-y

[98] Azcón R, Ambrosano E, Charest C. Nutrient acquisition in mycorrhizal lettuce plants under different phosphorus and nitrogen concentration. Plant Sci. 2003;165:1137–1145. doi: 10.1016/S0168-9452(03)00322-4

