# Supplementary figures and images for: Effects of growth promoting microorganisms on tomato seedlings growing in different media conditions
Source: PLoS One. 2021 Nov 3;16(11):e0259380. doi: 10.1371/journal.pone.0259380 (PMC8565787; doi:10.1371/journal.pone.0259380)

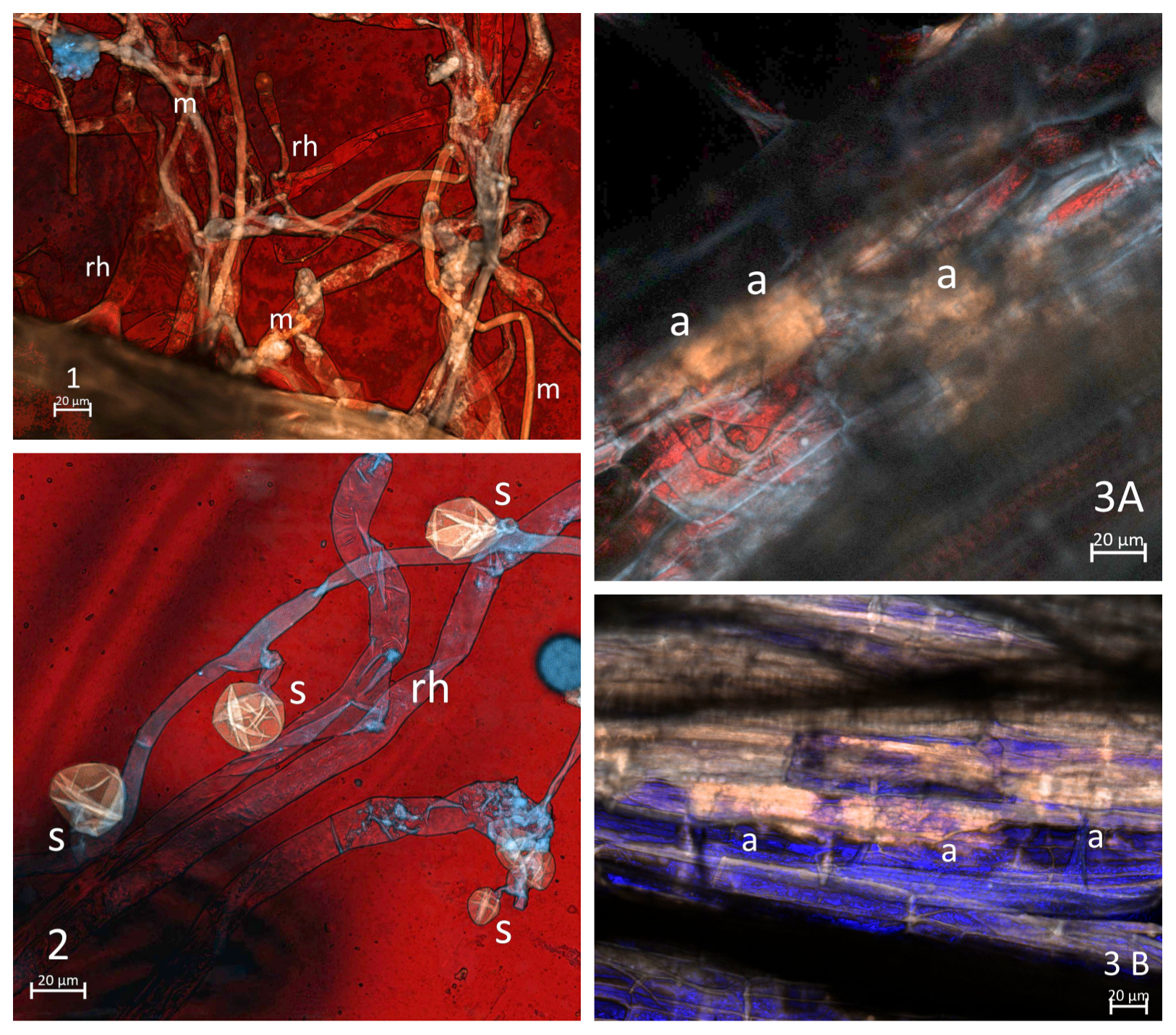

Supplement: S1 Fig — The soil with higher organic matter has showed better netting. Figure was taken in sample at AMF + AZ 100 treatment. 1_2. Development of AMF spores (s) in root hairs (rh) area was also detected after inoculation in the treatment with low organic matter content (AMF + AZ 50). This confirms ability of AMF to colonize plant roots and form the propagative structures for further substrate colonizing. 1_3 A, 1_3B. Set up of symbiosis between AMF and tomato plants was described on arbuscules (a) structures found in root tissues. The treatments with peat showed higher levels of arbuscules abundance. Both figures show the symbiosis created in sand:peat substrate 50:50, also (AMF + AZ 50). Bar = 20 μm. (TIF) [file pone.0259380.s001.tif]

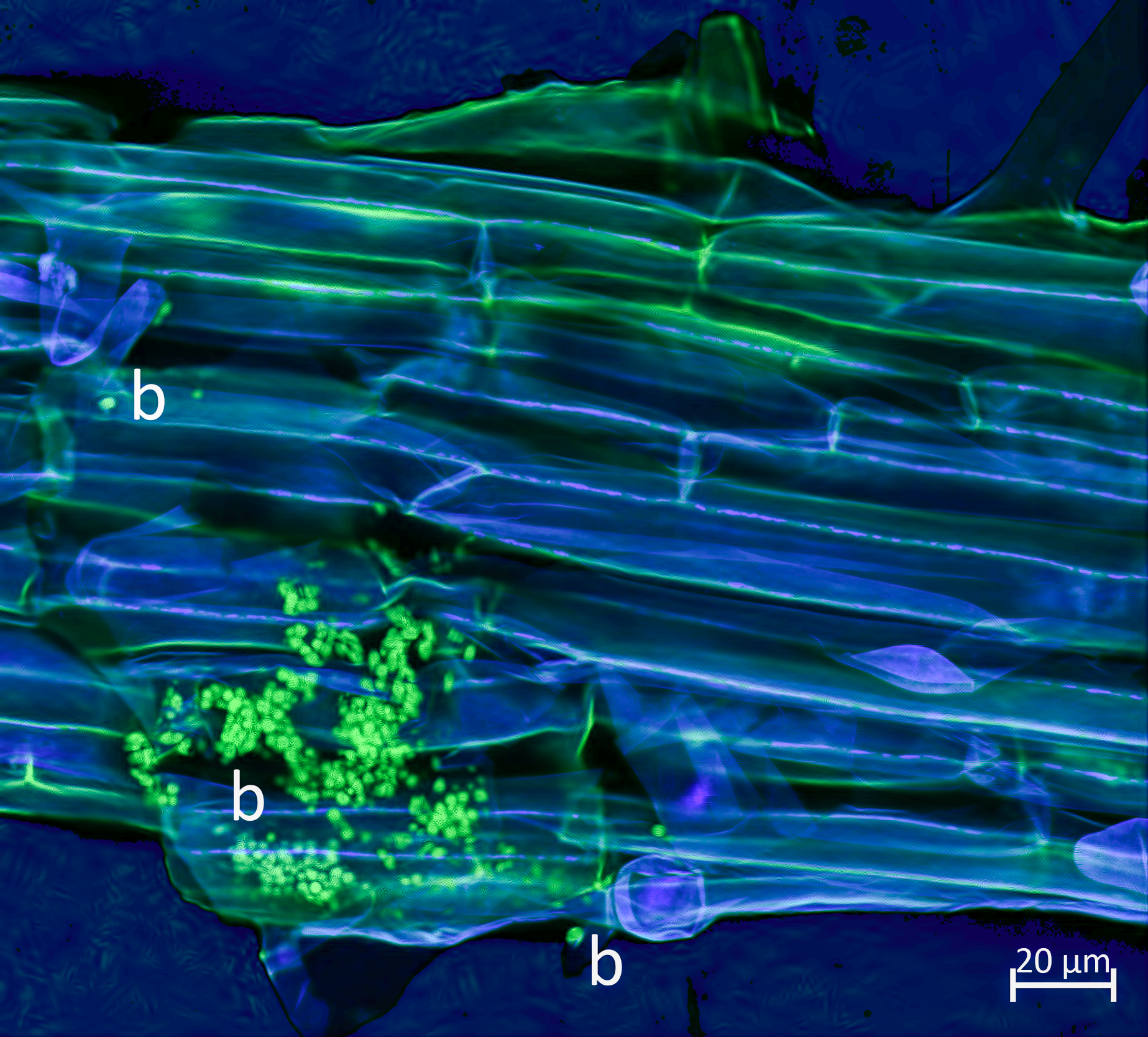

Supplement: S2 Fig — In sterile conditions of substrate were found only these bacterial colonies as abundant (treatment AMF + AZ 70). Bar = 20 μm. (TIF) [file pone.0259380.s002.tif]

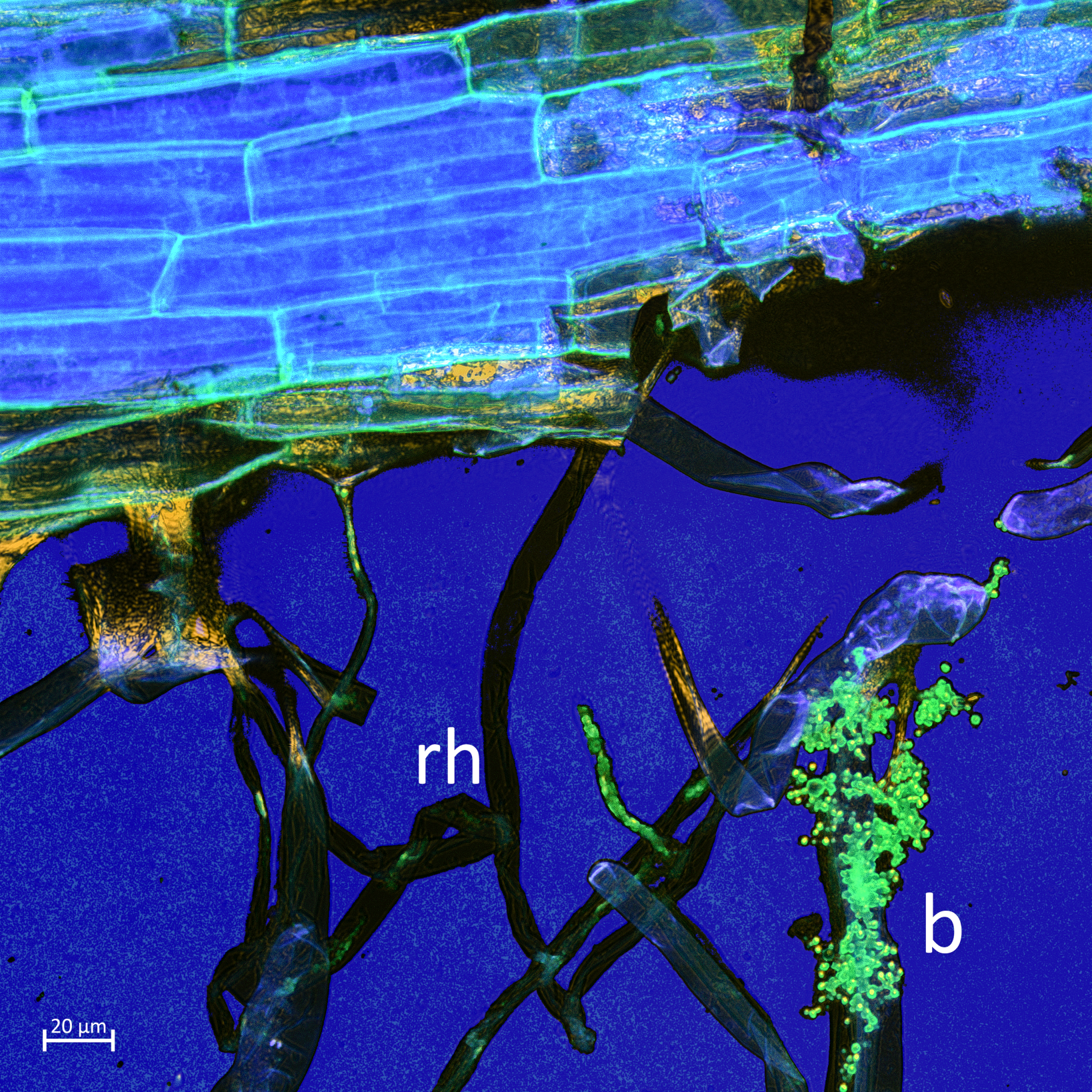

Supplement: S3 Fig — In sterile conditions of substrate were found only these bacterial colonies as abundant (treatment: AMF + AZ 100). Bar = 20 μm. (TIF) [file pone.0259380.s003.tif]
