# Supplementary material for: Effects of growth promoting microorganisms on tomato seedlings growing in different media conditions
Source: PLoS One. 2021 Nov 3;16(11):e0259380. doi: 10.1371/journal.pone.0259380 (PMC8565787; doi:10.1371/journal.pone.0259380)
Supplement: Attachment — Submitted filename: response to reviewers.docx [file pone.0259380.s004.docx]

**Plos One**

Response to the reviewers

**Dr.** **Umakanta Sarker**

October 1^st^, 2021 Lednice

Dear editor,

I am writing in matter of re-submitting our manuscript newly entitled “Effects of growth promoting microorganisms on tomato seedlings growing in different media conditions”, authors Robert Pokluda, Lucia Ragasová, Miloš Jurica, Andrzej Kalisz, Monika Komorowska, Marcin Niemiec, and Agnieszka Sekara after revision required by reviewers.

We enclosed all demanded files with an appropriate change:

1. Rebuttal letter
2. Marked copy of MS with highlighted changes
3. Unmarked revised MS
4. Substituted Pictures No 1,2,3A,3B by Picture 1 merging them together
5. Renamed Pictures 2 and 3 (formerly 4A,4B)

List of references was updated by excluding all, which were relevant to the excluded part of text in manuscript, and adding 1 reference, according to the reviewer demand.

Reviewers' comments answers:

Reviewer #1: Materials and methods are well defined and seems reproducible.
Consider modifying title as "Effects of growth promoting microorganisms on tomato seedlings growing in different media conditions".

Answer: Title changed according reviewer

Introduction is too long, consider reducing it by 30% or three pages in total.

Answer: Length changed to the demanded size

Likewise, discussion is too long, consider reducing it significantly and make it crisp.
Answer: Discussion significantly shortened. MS was shortened by 10 pages, in total.

Combine picture 1-4 as a new Figure and update figure numbers

Answer: Pictures 1-4 merged, numbering updated

Reviewer #2: In this paper, the authors address a question of high interest for researchers studying the role of PGPM on tomato under different substrate conditions. The paper is correctly designed and carried out. Results are also consistent and very interesting. However, the current version must be revised at several points before it can be accepted. A list of the points to be addressed when revising the paper is provided below:

L35: Consider replacing “This review (Borelli et al., 2021)…” by “The review of Borelli et al. (2021)…”

Answer: excluded by shortening of Introduction

L73: Replace “They” with “AMF”

Answer: replaced

L80-81: Consider re-writing the sentence in a different way. You can state that “Merrouche et al. (2017) have previously reported that…..’’

Answer: L62 – changed according to reviewer

L119: Replace “little understood” by “poorly understood”

Answer: L78 – changed according to reviewer

L120: Replace tomato’s scientific name with the updated one

Answer: valid name added to the Methodology L 107

L137: Is there any reference available which can support that easily degradable soil will be tolerated by plants when PGPM will be applied. Consider describing in more detail that PGPM can increase the tolerance of X number of soil stresses, so tomatoes will benefit from PGPM when cultivated under easily degradable soil.

Answer: L86 ref. Inui Kishi et al. (2017) added

L460-461: Please re-write the sentence in a more understandable way.
Answer: L413-415, changed to clear statement

L573-574: As the referred element (Phosphorus) was referred at the previous page, consider refer again as P, and not as ‘this element’.
Answer: changed this element to P

L629: Re-locate the words of the sentence to ‘both necessary and potentially toxic for plants’
Answer: L580 changed

L650: was your previous researched published? Can you provide the relevant reference?
Answer: By shortening of Discussion was this part excluded

L765-766: Can you provide reference for the sentence ‘Moreover, plants …deficit conditons’?

Answer: By shortening of Discussion was relevant part excluded

Thank you for,

Yours sincerely

Prof.Ing.Robert Pokluda, Ph.D. and co-authors
