# Supplementary material for: Effects of growth promoting microorganisms on tomato seedlings growing in different media conditions
Source: PLoS One. 2021 Nov 3;16(11):e0259380. doi: 10.1371/journal.pone.0259380 (PMC8565787; doi:10.1371/journal.pone.0259380)
Supplement: Attachment — Submitted filename: review response.docx [file pone.0259380.s005.docx]

**Response to reviewers**	October 17^th^ 2021

According to the demands, there were done all changes, as described below.

ACADEMIC EDITOR:

The authors addressed all the comments raised by reviewers and both reviewers are accepted the MS. Now, the manuscript improved substantially. However, before its acceptance, the authors should address these issues again with minor revision.

-There are several missing spacing between numbers and °C, before and after the symbol “+” (numerous), and “±”, etc. throughout the whole MS including figure and Table values and captions.  Check carefully and addressed accordingly.

ANSWER: corrected spacing by all symbols, units

-Follow the citation style of PLOS one in the text and reference chapter.

References are listed at the end of the manuscript and numbered in the order that they appear in the text. In the text, cite the reference number in square brackets (e.g., “We used the techniques developed by our colleagues [19] to analyze the data”). PLOS uses the numbered citation (citation-sequence) method and first six authors, et al.

ANSWER: corrected to rule 6 authors max, citation style corrected

Line 109: Change “120°C” to “120 °C”.

ANSWER: corrected

Line 190-191: Change “The DPPH scavenging activity against 2,2-diphenyl-1-picrylhydrazyl (DPPH radical) was determined in plant samples.” to “The antioxidant activity was determined in plant samples following DPPH radical (2,2-diphenyl-1-picrylhydrazyl) scavenging method (add a reference here. the author may cite Doi.10.1038/s41598-021-91157-8).”.

ANSWER: corrected

Line 254: Table 2: Change “mMol” to “mM”.

ANSWER: corrected

-Discussion needs to be improved with the mechanistic explanation of the results citing relevant references.

Line 657: To explain the reason for enhanced polyphenol synthesis add the sentences at the end of the sentence “-----(Sharma et al., 2019).” “Abiotic stresses create osmotic stress (doi.10.3389/fpls.2020.559876), oxidative damage (doi.10.1007/s12010-018-2784-5), and reactive oxygen species (ROS) (doi.10.1038/s41598-018-34944-0), that lead to numerous physio-molecular changes, including a decrease in photosynthetic activities (doi.10.1016/j.foodchem.2018.01.097), DNA, protein and membrane damages, and nutritional imbalance in plants (doi.10.1371/journal.pone.0206388) and ultimately affect plant growth and productivity (doi.10.1002/jsfa.9423). Nevertheless, to adjust stress, stress-induced plant evolved mechanisms to enhance the concentration of the majority of polyphenols (doi.10.1038/s41598-018-30897-6; doi.10.1186/s12870-018-1484-1) and detoxify the ROS."

Line 664: Phenolic compounds have high antioxidant activity (add a reference here. the author may cite doi.10.3389/fnut.2020.587257) that can scavenge reactive oxygen species (add a reference here. the author may cite (doi.10.1186/s12870-020-02780-y),

Line 671: Change “elements mentioned in the present study” to “elements mentioned in the present study were corroborative to the previous findings (add a reference here. the author may cite doi.10.1038/s41598-020-71714-3)”.

ANSWER: corrected

Yours faithfully

Robert Pokluda
